# Polymorph selection towards photocatalytic gaseous CO_2_ hydrogenation

**DOI:** 10.1038/s41467-019-10524-2

**Published:** 2019-06-07

**Authors:** Tingjiang Yan, Lu Wang, Yan Liang, Meysam Makaremi, Thomas E. Wood, Ying Dai, Baibiao Huang, Feysal M. Ali, Yuchan Dong, Geoffrey A. Ozin

**Affiliations:** 1 The Key Laboratory of Life-Organic Analysis, College of Chemistry and Chemical Engineering, Qufu Normal University, Qufu, Shandong 273165 P. R. China; 2 Materials Chemistry and Nanochemistry Research Group, Solar Fuels Cluster, Department of Chemistry, University of Toronto, 80 St. George Street, Toronto, ON M5S 3H6 Canada; 3 School of Physics, State Key Laboratory of Crystal Materials, Shandong University, Jinan, Shandong 250100 P. R. China

**Keywords:** Heterogeneous catalysis, Photocatalysis, Nanoparticles

## Abstract

Titanium dioxide is the only known material that can enable gas-phase CO_2_ photocatalysis in its anatase and rutile polymorphic forms. Materials engineering of polymorphism provides a useful strategy for optimizing the performance metrics of a photocatalyst. In this paper, it is shown that the less well known rhombohedral polymorph of indium sesquioxide, like its well-documented cubic polymorph, is a CO_2_ hydrogenation photocatalyst for the production of CH_3_OH and CO. Significantly, the rhombohedral polymorph exhibits higher activity, superior stability and improved selectivity towards CH_3_OH over CO. These gains in catalyst performance originate in the enhanced acidity and basicity of surface frustrated Lewis pairs in the rhombohedral form.

## Abstract (web summary)

Polymorphs, compounds with identical chemical stoichiometries yet different atomic configurations, expand the range of potential chemical properties and new applications. Here, authors show rhombohedral indium oxides to be highly active and selective for photocatalytic CO_2_ hydrogenation.

## Introduction

A breakthrough in CO_2_ photocatalysis often begins with materials discovery, and then, the challenges of optimizing its performance metrics by materials engineering follows. Through human intelligence and experiential learning, complemented by artificial intelligence and machine learning, one can hone the chemical and physical properties of a material to achieve the desired catalyst optimization for a targeted technology.

In the case of gas-phase heterogeneous hydrogenation of CO_2_ to chemicals and fuels, performance optimization usually involves fine tuning the chemical and physical properties of the (photo)catalyst^1–3^. This can be achieved by doping^4,5^, isomorphous and aliovalent substitution^6,7^, size control^8,9^, morphology changes^10,11^, heterostructuring^12–14^, and support and promoter effects^15–17^.

In this endeavor, a strategy rarely employed in catalyst optimization is polymorph selection, whereby the crystal structure of the catalyst material is changed, whereas its composition is retained. The challenge today is moving beyond titanium dioxide anatase and rutile polymorphs and discovering other compositions that exhibit polymorphism and enable CO_2_ (photo)catalysis.

Indium-based semiconductor materials have been reported as promising photocatalysts in terms of their geometric structures and electronic configurations, which can possibly enhance the mobility and separation efficiency of charge carriers and improve photocatalytic activity^18,19^. Numerous attempts have been made in recent years to develop various indium-based photocatalysts, such as oxides or mixed oxides^20,21^, binary and ternary sulfides^22,23^, hydroxides and oxyhydroxides^24,25^, In-MOF^26^, and so on. Among them, indium oxide (In_2_O_3_) is an n-type semiconductor with mainly two polymorphic phases, cubic and metastable rhombohedral phases^27^. Pan et al.^28^ and co-workers have demonstrated that cubic In_2_O_3_ nanobelts coated by carbon layer showed highly enhanced photocatalytic reduction of CO_2_ to CO and CH_4_ in aqueous solution with Pt as co-catalyst. Our group has reported that the cubic indium oxide nanocrystals with surface defects in the form of oxygen vacancies and hydroxyl groups, denoted as In_2_O_3-x_(OH)_y_, can be utilized as an active catalyst towards photocatalytic gaseous CO_2_ hydrogenation owing to advantageous surface, optical, and electronic properties^29^. The CO_2_ hydrogenation performance of the cubic In_2_O_3-x_(OH)_y_ polymorph can be further boosted by assembling nanocrystals into rod-like superstructures, which gives rise to an improved conversion rate for the reverse water gas shift reaction and a champion rate for solar methanol production at atmospheric pressure^10,30^.

Herein, we have discovered that the rhombohedral form of indium oxide, like its cubic polymorph, is a highly active and selective heterogeneous (photo)catalyst for hydrogenating gaseous CO_2_ to CO and CH_3_OH. Significantly, the rhombohedral polymorph outperforms the cubic polymorph in terms of its catalytic activity, long-term stability, and selectivity towards CH_3_OH. These performance enhancements stem from an increase in the acidity and basicity of surface frustrated Lewis pairs (SFLP) in the rhombohedral compared with the cubic polymorph.

## Results

### Synthesis and structural characterizations of rhombohedral In_2_O_3-x_(OH)_y_

**Fig. 1:**
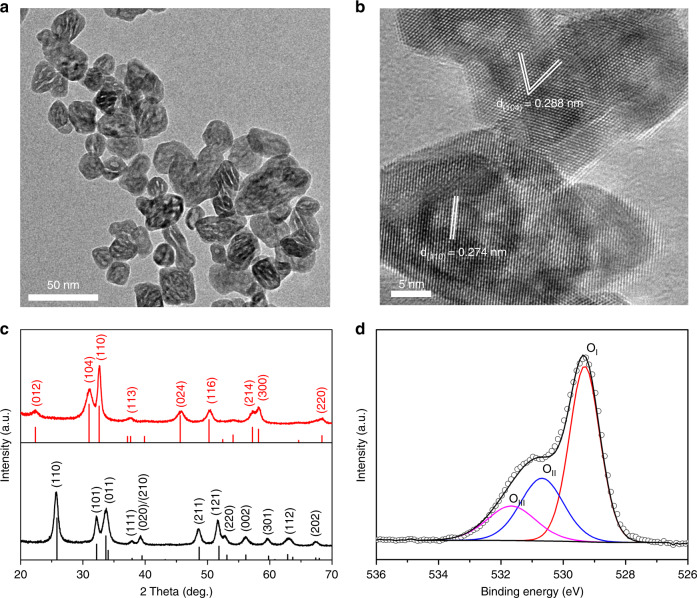
Structural characterizations of InOOH precursor and rhombohedral In_2_O_3-x_(OH)_y_ nanocrystals. **a** TEM image of rh-In_2_O_3-x_(OH)_y_ nanocrystals. **b** HRTEM image of rh-In_2_O_3-x_(OH)_y_ nanocrystals. **c** PXRD patterns of InOOH precursor (black) and rh-In_2_O_3-x_(OH)_y_ nanocrystals (red). **d** High-resolution O 1 s core level XPS spectra for rh-In_2_O_3-x_(OH)_y_ nanocrystals

The rhombohedral In_2_O_3-x_(OH)_y_ nanocrystals (annealing at 350 °C and denoted as rh-In_2_O_3-x_(OH)_y_) were synthesized via a thermal dehydration of an InOOH precursor, which was initially synthesized from a simple solvothermal system^31^. The obtained InOOH precursor is composed of monodispersed nanoparticles with the size of ~ 20 nm (Supplementary Fig. 1). After thermal treatment at 350 °C, the resultant rh-In_2_O_3-x_(OH)_y_ nanocrystals maintained a similar particle size and exhibited a walnut shell-like morphology, which contained considerable nanopores in each nanocrystal (Fig. 1a). Such unique morphology could be caused by the loss of water from the lattice of the InOOH nanocrystal, which was confirmed by thermogravimetry (TG) and derivative thermogravimetry (DTG) analysis (Supplementary Fig. 2). In this case, the presence of these nanopores did not contribute much to the specific surface area of rh-In_2_O_3-x_(OH)_y_. As a result, the as-prepared rh-In_2_O_3-x_(OH)_y_ has a specific surface area of 56 m^2^ g^−1^ and a pore size of 11 nm (Supplementary Fig. 3). The high-resolution transmission electron microscopy (HRTEM) images (Fig. 1b and Supplementary Fig. 4) indicate well-defined lattice fringes with inter-planar distances of 0.274 nm and 0.288 nm, which correspond to the (110) and (104) facets of rhombohedral In_2_O_3_, respectively. The powder X-ray diffraction (PXRD) patterns confirm that the precursor is orthorhombic InOOH, and leads to the formation of the corundum structure type of In_2_O_3_ (Fig. 1c). An obvious shift in binding energy was observed from the high-resolution X-ray photoelectron spectroscopy (XPS) of In 3d core level spectra for samples before and after annealing, indicating the conversion from InOOH to In_2_O_3-x_(OH)_y_ (Supplementary Fig. 5). The O 1 s core level XPS spectra (Fig. 1d) could be fitted into three peaks at 529.3 eV, 530.7 eV, and 531.8 eV, which can be assigned to oxides, oxygen vacancies, and hydroxyl groups, respectively^29^. The existence of oxygen vacancies is further evidenced by a strong diagnostic luminescent peak centered at ca. 500 nm in the PL spectrum (Supplementary Fig. 6). These results confirm the formation of oxygen vacancies as well as the coexistence of oxides, vacancies, and hydroxyl groups in rh-In_2_O_3-x_(OH)_y_, which play a key role in the formation of SFLP^32–34^.

### CO_2_ hydrogenation performance

**Fig. 2:**
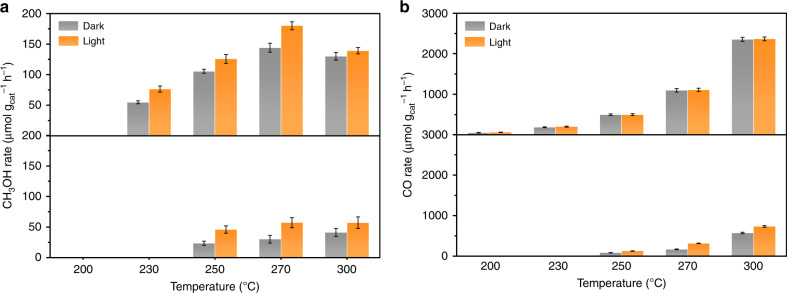
Catalytic performance of rh-In_2_O_3-x_(OH)_y_ (up) and c-In_2_O_3-x_(OH)_y_ (down). **a** CH_3_OH production rate at different reaction temperatures with and without solar irradiation. **b** CO production rate at different reaction temperatures with and without solar irradiation

The catalytic properties of the as-obtained rh-In_2_O_3-x_(OH)_y_ nanocrystals toward CO_2_ hydrogenation with and without solar irradiation were evaluated in a flow reactor at different temperatures, under atmospheric pressure with a mixed feed gas of CO_2_ and H_2_ (H_2_: CO_2_ = 3: 1) (Fig. 2a, b). To investigate the polymorph effect of indium oxide on CO_2_ hydrogenation performance, the cubic In_2_O_3-x_(OH)_y_ nanocrystals (annealing at 300 °C and denoted as c-In_2_O_3-x_(OH)_y_) with similar porous morphology and crystalline size (Supplementary Fig. 7) were synthesized based on previous studies^29^ and utilized as a reference material for comparison. The catalytic performance of the selected photocatalysts was tested from 200 °C to 300 °C. Only the as-prepared rh-In_2_O_3-x_(OH)_y_ was found to be able to catalyze the reverse water gas shift (RWGS) reaction at 200 °C with a CO rate of 51 μmol g_cat_^−1^ h^−1^ and 60 μmol g_cat_^−1^ h^−1^ for dark and light conditions, respectively. When the reaction temperature was increased to 230 °C, the rh-In_2_O_3-x_(OH)_y_ showed an increased CO formation rate of 185 μmol g_cat_^−1^ h^−1^ (in dark) and 201 μmol g_cat_^−1^ h^−1^ (in light). Furthermore, at this specific temperature, only the rh-In_2_O_3-x_(OH)_y_ was found to be capable of synthesizing CH_3_OH with a rate of 55 μmol g_cat_^−1^ h^−1^ (in dark) and 76 μmol g_cat_^−1^ h^−1^ (in light).

When the temperature was increased to 250 °C, the rh-In_2_O_3-x_(OH)_y_ exhibited a CH_3_OH rate of 105 μmol g_cat_^−1^ h^−1^ (in dark) and 126 μmol g_cat_^−1^ h^−1^ (in light). Thereafter, increasing the reaction temperature to 270 °C resulted in a CH_3_OH formation rate of 180 μmol g_cat_^−1^ h^−1^ and 144 μmol g_cat_^−1^ h^−1^ with and without light irradiation. Such a solar powered CH_3_OH rate of 180 μmol g_cat_^−1^ h^−1^ is a performance record^35–38^ and about two times higher than the best reported solar CH_3_OH maker (c-In_2_O_3-x_(OH)_y_ nanorods)^30^ and 3.5 times higher than the reference c-In_2_O_3-x_(OH)_y_ nanocrystals at similar reaction conditions. If catalytic performance is normalized on the basis of the specific surface area, the rh-In_2_O_3-x_(OH)_y_ would show a solar CH_3_OH rate of 3.2 μmol h^−1^ m^−2^, which is ~ 5.8 times higher than that of c-In_2_O_3-x_(OH)_y_ nanorods, or 8.6 times higher than that of c-In_2_O_3-x_(OH)_y_ nanocrystals. A control experiment shows that the CH_3_OH selectivity of the commercial thermal catalyst (alumina supported copper zinc oxide) under the same photothermal reaction conditions and ambient pressure is ∼ 0.4 %, which is 32 times lower than rh-In_2_O_3-x_(OH)_y_. Although the CH_3_OH productivity of rh-In_2_O_3-x_(OH)_y_ under high pressure needs further research, the rh-In_2_O_3-x_(OH)_y_ shows great potential for the future development of a solar fuels economy. Compared with traditional thermocatalytic technologies, solar-driven photocatalytic and photothermal catalytic CO_2_ hydrogenation are proving to be promising strategies as they enable the utilization of abundant and clean solar energy, and during the catalytic process the photoexcitation of electrons into higher-energy states can lower the energy barrier of reaction^39^.

Owing to the exothermic nature of the CH_3_OH synthesis from H_2_-CO_2_, the CH_3_OH rate at 300 °C decreases slightly to 139 μmol g_cat_^−1^ h^−1^ (in light) and 130 μmol g_cat_^−1^ h^−1^ (in dark). The enhancement of the CH_3_OH rate with light irradiation can be attributed to the lower activation energy of the photocatalytic process as compared with the thermochemical process^40^. The Arrhenius plots over rh-In_2_O_3-x_(OH)_y_ (Supplementary Fig. 8a) yield the apparent activation energy for CH_3_OH production reaction, photocatalytically, at 38.4 kJ mol^−1^, much smaller than 59.8 kJ mol^−1^ for the thermochemical reaction. Moreover, the activation energy for CH_3_OH production over rh-In_2_O_3-x_(OH)_y_ is much lower than that of c-In_2_O_3-x_(OH)_y_ under the same conditions (Supplementary Fig. 8b) and also lower than the reported value (103 kJ mol^−1^) for cubic indium oxide under dark condition^41^.

To further support that CH_3_OH production mainly proceeds through a photochemistry process, we also examined the dependence of CH_3_OH rate on the wavelength of incident light. The results showed that the CH_3_OH rate decreased with decreasing wavelength of the light (Supplementary Fig. 9), which matches well with the optical absorption spectra of rh-In_2_O_3-x_(OH)_y_. The photo-enhancement for CH_3_OH production almost disappeared when a 500 nm cutoff filter near the absorption edge was applied for the same light intensity.

Conversely, owing to the endothermic nature of the RWGS reaction, the CO rate is dramatically enhanced with increasing temperature and reaches about 2.4 mmol g_cat_^−1^ h^−1^ at 300 °C. Such a CO formation rate is comparable to some of the most active noble metal decorated catalysts^13,42^ and ~ 3.2 times higher than that of the reference c-In_2_O_3-x_(OH)_y_. Unlike CH_3_OH performance, which can be enhanced by the light irradiation, CO performance was only enhanced by ∼ 0.5 % under light irradiation (activation energy of ∼ 84.8 kJ mol^−1^ for both light and dark), which results in an enhanced solar CH_3_OH selectivity (Supplementary Fig. 10). The distinct light-dependent trend reveals that the rate determining step for the production of CO appears to occur mainly by a thermochemical pathway in the electronic ground state, whereas the production of methanol has a contributing photochemical pathway involving electrons and holes in the electronic excited state thereby enhancing the activity of the SFLP. Moreover, to confirm the veracity of the CO and CH_3_OH products from CO_2_, isotope tracing experiments were conducted, where the ^13^CO_2_ feedstock was utilized and the product gases were analyzed by gas chromatography–mass spectrometry (GC-MS), which confirmed the presence of ^13^CO and ^13^CH_3_OH and the verity of the CO_2_ derived products (Supplementary Fig. 11).

### Tuning of SFLP toward CO_2_ hydrogenation on rhombohedral In_2_O_3-x_(OH)_y_

The structural parameters including concentration of oxygen vacancies and hydroxides are of great importance in forming the SFLP, and subsequently influence the photocatalytic performance. Accordingly, a series of rh-In_2_O_3-x_(OH)_y_ nanocrystals were synthesized from the InOOH precursor with different annealing temperatures of 250, 300, 350, and 400 °C, and denoted as rh-250, rh-300, rh-350, and rh-400, respectively. PXRD patterns are found to be similar for samples annealed above 300 °C and could be assigned to the corundum structure type of In_2_O_3_ (Supplementary Fig. 12). Interestingly, the sample annealed at 250 °C exhibited a similar PXRD pattern to the InOOH precursor, which indicated a similar structure to InOOH, but with more oxygen vacancies and less hydroxide groups. The grain sizes for the prepared rh-In_2_O_3-x_(OH)_y_ nanocrystals showed a gradual decrease with elevated annealing temperatures, along with more nanopores generated during the dehydroxylation reaction (Supplementary Figs. 13 and 14). The specific surface areas of the rh-In_2_O_3-x_(OH)_y_ nanocrystals are slightly larger than the precursor InOOH, but are lower than the c-In_2_O_3-x_(OH)_y_. All rh-In_2_O_3-x_(OH)_y_ samples show the same In(III) oxidation state, and the concentration of oxygen vacancies increases with increasing annealing temperatures, whereas the concentration of hydroxides decreases simultaneously (Supplementary Fig. 15). The band-edge absorption for all rh-In_2_O_3-x_(OH)_y_ nanocrystals and c-In_2_O_3-x_(OH)_y_ is located at ~ 450 nm, indicating similar electronic band structures (Supplementary Fig. 16).

**Fig. 3:**
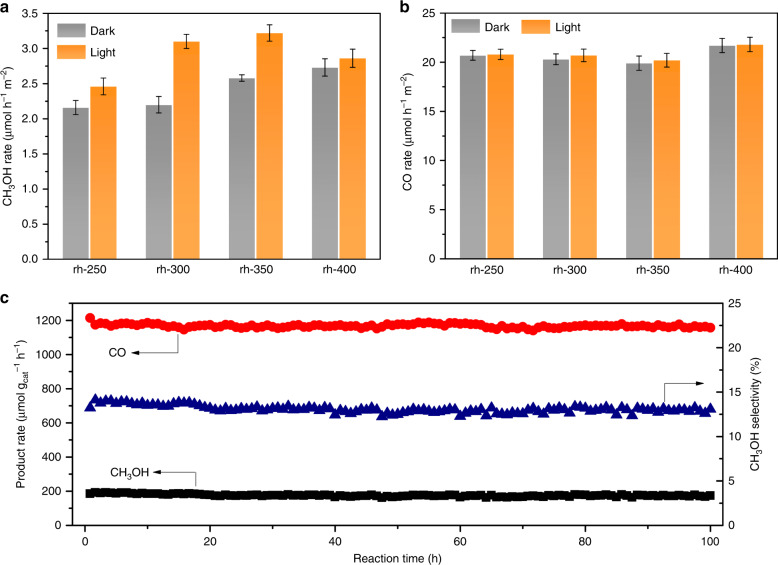
Catalytic performance of various rh-In_2_O_3-x_(OH)_y_ nanocrystals. **a** Normalized CH_3_OH production rate at 270 °C with and without light irradiation. **b** Normalized CO production rate at 270 °C with and without light irradiation. **c** Long-term (100 h) catalytic stability of rh-In_2_O_3-x_(OH)_y_ nanocrystals (rh-350) in catalyzing hydrogenation of CO_2_ with light irradiation; reaction condition: 270 °C, 6 ml min^−1^ H_2_ and 2 ml min^−1^ CO_2_

The normalized catalytic activity for the various surface tuned rh-In_2_O_3-x_(OH)_y_ nanocrystals toward CO_2_ hydrogenation, at 270 °C and 300 °C, with and without illumination, are shown in Fig. 3a, b, Supplementary Fig. 17 and summarized in Table 1. Although the rh-250 still showed similar PXRD patterns to InOOH, the thermal treatment was able to remove some lattice oxygen as well as hydroxide groups, which may result in the formation of SFLP and exhibit catalytic performance toward CO_2_ hydrogenation. Other than the rh-250, the as-prepared rh-300, rh-350 and rh-400 exhibited diagnostic PXRD patterns, which can be assigned to the rhombohedral In_2_O_3_ structure with major exposed facets of (110) and (104) (Supplementary Fig. 18). The calculated grain size of rh-In_2_O_3-x_(OH)_y_ were ~ 11.5 ± 1.5 nm for all selected samples. All samples exhibited similar structural and morphological properties as well as photocatalytic performance, whereas the rh-350 showed the best catalytic performance.

**Table 1:** Summary of properties of various In_2_O_3-x_(OH)_y_ samples

Sample	D^a^	A^b^	E_g_^c^	O_v_^d^	OH^e^	R_CO_^f^	R_Methanol_^g^
rh-250	13	44	3.15	20.01	31.87	55.57	2.47
rh-300	13	54	3.01	21.35	20.64	44.87	3.10
rh-350	11	56	2.89	26.88	17.96	42.14	3.21
rh-400	10	51	2.88	30.00	16.40	38.31	2.86
c-In_2_O_3-x_(OH)_y_	9.5	138	2.89	23.37	19.09	5.31	0.37

^a^Grain size calculated from PXRD patterns (nm)

^b^Specific surface area obtained from BET measurement (m^2^ g^−1^)

^c^Band gap energy (eV) calculated by fitting the reflectance spectra using K–M theory

^d^Concentration of oxygen vacancies calculated from XPS (at. %)

^e^Concentration of hydroxide groups calculated from XPS (at. %)

^f^Normalized CO rate with solar irradiation obtained at 300 °C (μmol h^−1^ m^−2^)

^g^Normalized CH_3_OH rate with solar irradiation obtained at 270 °C (μmol h^−1^ m^−2^)

The CO_2_ adsorption properties were further investigated by CO_2_-TPD experiments. As shown in Supplementary Fig. 19 and Table 1, the desorption temperature of CO_2_ molecules on c-In_2_O_3-x_(OH)_y_ (~ 157 °C) is higher than that of all rhombohedral samples (~ 140 °C), indicating that the CO_2_ molecules are more tightly absorbed on the surface of c-In_2_O_3-x_(OH)_y_. Moreover, the c-In_2_O_3-x_(OH)_y_ shows a larger peak area, reflecting an enhanced CO_2_ adsorption. The reason for the higher CO_2_ adsorption capacity of c-In_2_O_3-x_(OH)_y_ may simply be due to its larger surface area as compared with rhombohedral samples, so that the c-In_2_O_3-x_(OH)_y_ could provide a larger population of capture sites for CO_2_. Even so, the enhanced CO_2_ adsorption capacity of c-In_2_O_3-x_(OH)_y_ cannot contribute much to its photocatalytic CO_2_ hydrogenation performance as the activity of rhombohedral samples greatly exceeded that of c-In_2_O_3-x_(OH)_y_, and indicates the SFLP site served as the catalytically active site for CO_2_ hydrogenation.

As SFLP can be considered as the active sites for the catalytic performance, the thermal treatment at 350 °C can efficiently remove lattice oxygen as well as hydroxide group to tune the surface into a ratio of 1:0.67 between oxygen vacancy and hydroxide group, which results in the optimal composition among all the samples tested. This can be further confirmed by the decreased activity of a H_2_ treated rh-350 sample, which possessed relatively more oxygen vacancies and less hydroxide groups (Supplementary Fig. 20). Furthermore, as compared with CO, the production of methanol was significantly improved when irradiating with light, and exhibited an enhanced methanol selectivity as well.

Owing to its resulting best photocatalytic performance, the rh-350 was selected for further long-term stability testing at 270 °C under atmospheric pressure with light irradiation. As shown in Fig. 3c, excellent stability resulted, with no significant change in production rate as well as selectivity in rh-350 over more than 100 h of continuous testing. As a result, methanol selectivity was maintained at 13% throughout the test, with the methanol rate of 170 μmol g_cat_^−1^ h^−1^ and CO rate of 1150 μmol g_cat_^−1^ h^−1^. Moreover, the used catalyst was also evaluated by XPS, PXRD, TEM, and no obvious oxidation state and structural changes were observed (Supplementary Figs. 21–23).

### The SFLP on rhombohedral In_2_O_3-x_(OH)_y_

The surface vacancy breaks the stable Lewis acid–base adjuncts (bridging In–O or In–OH), which can then create novel surface Lewis acidic sites, and the nearby surface hydroxide groups can function as the Lewis basic sites. As such, the strong charge difference between the resulting unsaturated In atom and the hydroxide group can form the SFLP on the single component metal oxide, which could further activate the small H_2_ molecules. The determination of the presence of this SFLP on defect laden rh-In_2_O_3-x_(OH)_y_ was performed as follows.

**Fig. 4:**
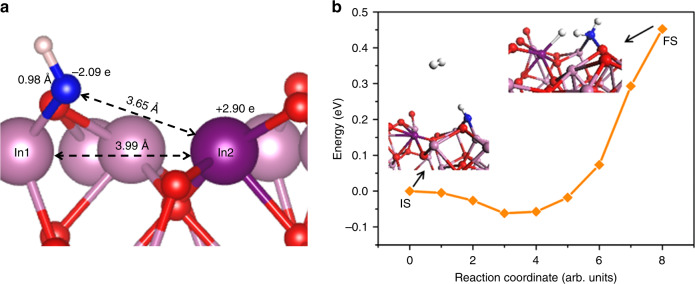
Surface frustrated Lewis pairs on rh-In_2_O_3-x_(OH)_y_. **a** Side view of optimized configuration for (110) rh-In_2_O_3-x_(OH)_y_. **b** Reaction pathway and energy barrier of H_2_ dissociation on (110) rh-In_2_O_3-x_(OH)_y_. White, pink, red, purple, and blue spheres represent H, In, O, Lewis pair In, and Lewis pair O atoms, respectively

Density functional theory (DFT) simulations were carried out to investigate the properties of the rhombohedral In_2_O_3_ nanocrystal. Based on PXRD patterns and HRTEM images, both of the major facets, (110) and (104), were used for the determination of the removal of lattice oxygen and addition of OH (as hydroxide). The (110) facet has been chosen for further calculation and explanation owing to the much stronger Bader charge of the present SFLP. To investigate this further, the bulk rhombohedral In_2_O_3_ structure was cut along the direction of (110) and generated the corresponding surface. As shown in Supplementary Fig. 24, possible vacancy sites were determined on the (110) rhombohedral In_2_O_3_ surface, and the formation energies of vacancies were calculated by using a known approach from the literature^40,43^. All atomic removal steps were found to be endothermic in which the adsorption energy would range from 4.15 (for site 1) to 4.94 eV (for site 3) (Supplementary Table 2). To simulate the rh-In_2_O_3-x_(OH)_y_ surfaces, which contain the OH group, the defected (110) rhombohedral In_2_O_3-x_ surface was used, which has the vacancies at site 1 and site 3 as the most and least favorable adsorption sites. Then the OH group was added to the defected surface at vacancy sites to form In_2_O_3-x_(OH)_y_, and the adsorption energy and charge transfer of binding were calculated (Supplementary Fig. 25). The amounts of total free energy change due to the binding of OH at site 1 and 3 of the defected surface are − 3.97 eV and − 4.30 eV, respectively, which indicate that the formation of hydroxylated nanostructures can be highly exothermic. Previous studies for the (111) surface of the c-In_2_O_3-x_(OH)_y_ nanostructure suggest that the O atom from the OH group and the near In atom (including + 1.66 e and − 1.50 e, respectively) can form an SFLP owing to the large charge difference^40^. Thus, Bader charge calculations were performed to probe the localized charges on surface constituents, showing that the related O and In pair involve atomic local charges of + 2.90 e and − 2.09 e, and + 2.90 e and − 2.03 e, for site 1 and site 3 of the rh-In_2_O_3-x_(OH)_y_ nanostructure (Fig. 4a), respectively. The larger Bader charge between the Lewis acid and Lewis base on rh-In_2_O_3-x_(OH)_y_ could be caused by the following contributing effects. (i) A larger geometrical distance between the active O atom and In atom (3.65 Å) than that of the c-In_2_O_3-x_(OH)_y_ (3.20 Å), and (ii) a higher coordination number of the active In atom (6 in (110) facets) in rh-In_2_O_3-x_(OH)_y_ than that in c-In_2_O_3-x_(OH)_y_ (4 in (111) facets). Similar to the (110) facets, the SFLP can also be constructed by removing surface oxygen atom and introducing a hydroxide group on (104) facets of rhombohedral In_2_O_3_ (Supplementary Figs. 26 and 27). It reveals that the Lewis acidic In and Lewis basic O of the OH sites at the (104) surface possess Bader charges of + 1.61 e and − 1.12 e, respectively. In this scenario, the distance between Lewis acid and base is 3.75 Å.

The larger charge difference between the Lewis acid and Lewis base pairs in the (110) rh-In_2_O_3-x_(OH)_y_ structure compared with that of the (111) c-In_2_O_3-x_(OH)_y_ surface, envisages the rh-In_2_O_3-x_(OH)_y_ nanostructure would form more active Lewis acid–base pairs than the c-In_2_O_3-x_(OH) pair can muster, and therefore could strongly polarize H–H bonds and dissociate H_2_ molecules. This prediction can be evidenced by the optimized configuration of hydrogenated rh-In_2_O_3-x_(OH)_y_, in which the H–H distance of H_2_ is enlarged from 0.75 Å to 1.24 Å and the bond length of newly formed In–H and H–OH bonds are 1.86 and 1.07 Å, respectively (Supplementary Fig. 28). Furthermore, the relative activation energy barrier for H_2_ heterolysis over rh-In_2_O_3-x_(OH)_y_ further confirms that the strong SFLP could enable more efficient H_2_ dissociation. The calculation of reaction pathway and energy barrier of H_2_ dissociation shows that H_2_ dissociation on (110) rh-In_2_O_3-x_(OH)_y_ surface is endothermic with an activation energy barrier of 0.45 eV (Fig. 4b), which is much smaller than that (0.66 eV) on the (111) c-In_2_O_3-x_(OH)_y_ surface^40^. More recently, experimental evidence for heterolysis of H_2_ on the SFLP of rh-In_2_O_3-x_(OH)_y_ has also been observed by our group using a suite of five insightful spectroscopy probes including diffuse reflectance infrared Fourier-transform spectroscopy (DRIFTS), XPS, ^1^H solid state MAS NMR, EPR, and UV-Vis-NIR, which provides an in-depth understanding of how gaseous H_2_ interacts with nanostructured rh-In_2_O_3-x_(OH)_y_.

### Investigation of the CO_2_ hydrogenation pathway

**Fig. 5:**
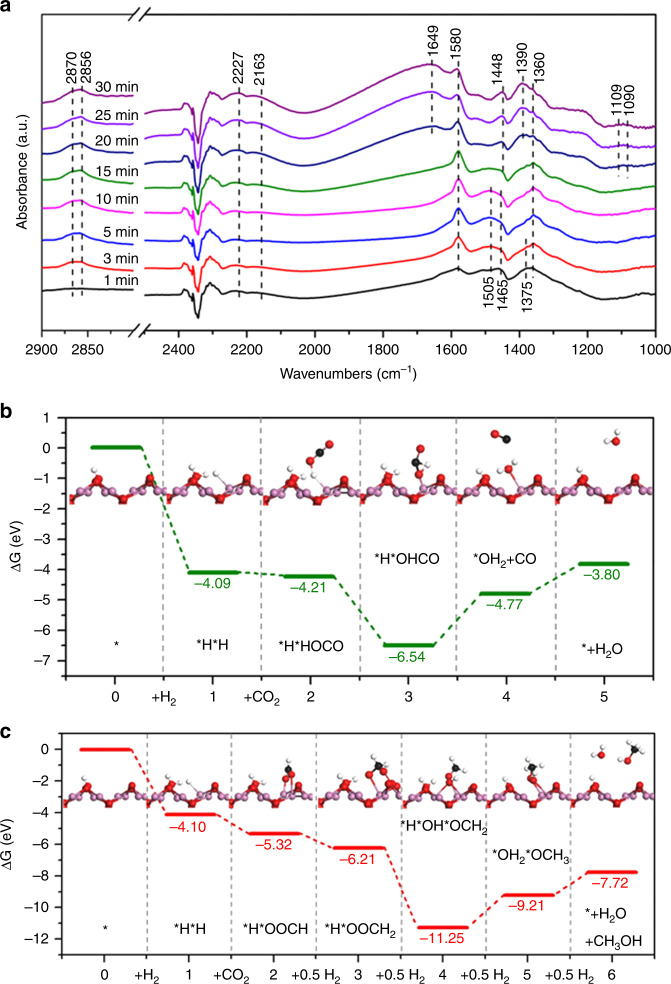
CO_2_ hydrogenation mechanism on rh-In_2_O_3-x_(OH)_y_ nanocrystals. **a** In situ DRIFTS spectra of surface species formed from CO_2_ hydrogenation. **b** Energy profiles for CO_2_ hydrogenation via the RWGS pathway. **c** Energy profiles for CO_2_ hydrogenation via the formate pathway. Insets are the corresponding structures of reaction intermediates. The zero energy corresponds to the total free energy of the rh-In_2_O_3-x_(OH)_y_ nanocrystal

In order to understand the catalytic pathway for CO_2_ hydrogenation on rh-In_2_O_3-x_(OH)_y_ nanocrystals, in situ DRIFTS measurements were performed in a flow cell under reaction operando conditions. In the initial reaction stage, two primary surface species were observed, as shown in Fig. 5a. The first kind of species with fingerprint modes at 1505, 1465, and 1375 cm^−1^ can be assigned to chemisorbed CO_2_ species including bicarbonate (HCO_3_^−^) and carbonate (CO_3_^2^^−^)^44–47^. The second kind of species signaled by fingerprint modes at 1580 and 1360 cm^−1^ can be attributed to the asymmetric and symmetric OCO stretching vibrations of adsorbed bidentate formate (HCOO*) species. This assignment is supported by two additional modes at 2870 cm^−1^ and 1390 cm^−1^ that are attributed to the stretching vibration ν(CH) and bending vibration δ(CH) of the same species^48–50^. Along with the CO_2_ hydrogenation the bicarbonate and carbonate species could readily be transformed to formate species, as evidenced by the decrease and disappearance of these bands. Apart from the formation of formate species, another important intermediate appears with diagnostic peaks at 2856, 1448, 1109, and 1090 cm^−1^ in the spectra that are assigned to methoxy (H_3_CO*)^48,49,51^. From these DRIFT results, CO_2_ hydrogenation over rh-In_2_O_3-x_(OH)_y_ may proceed via formate intermediates (Supplementary Fig. 29, formate pathway), which eventually produces CH_3_OH via the C-O bond cleavage and *HCO or *H_2_CO intermediates^48,52^. Simultaneously, we also observe the diagnostic vibrational modes of CO at bands 2227 and 2163 cm^−1^, and water at 1649 cm^−1^, indicative of another reaction pathway featuring a CO intermediate (Supplementary Fig. 29, RWGS pathway), which is produced from the RWGS reaction via carboxyl (*HOCO) intermediates^50,53^. However, such *HOCO intermediates are unstable and cannot be detected even at a low temperature (90 K)^54^. To corroborate these experimental observations, free energy profiles for CO_2_ hydrogenation via the proposed RWGS and formate pathways over rh-In_2_O_3-x_(OH)_y_ were calculated. The results suggest that via the RWGS pathway (Fig. 5b), except for product (CO, H_2_O) desorption, the preceding hydrogenation reactions steps 1–3 are all exothermic. Similarly, steps 1–4 also behave in an exergonic nature via the formate pathway (Fig. 5c). These are different from the previously reported c-In_2_O_3-x_(OH)_y_ surface^30,40^, resulting in the possibility of enhanced catalytic activity for hydrogenation of CO_2_. Notably, the smaller free energy barrier for the RWGS pathway is consistent with our experimental data of a faster CO production rate.

## Discussion

As the first report that an SFLP in cubic In_2_O_3-x_OH_y_^40^, denoted InOH•••In, can facilitate heterolysis of H_2_ to form InOH_2_^+^•••InH^−^, a number of chemical strategies have been devised to modify its Lewis acidity and Lewis basicity in order to tune its activity and selectivity in CO_2_ (photo)catalysis.

To amplify, following heterolysis of H_2_ on the SFLP, InOH•••In → InOH_2_^+^•••InH^−^, the proton bound to the hydroxide Lewis base and hydride bound to the coordinately unsaturated indium can subsequently react with CO_2_ to form CO in an endothermic reverse water gas shift reaction (Eq. 1):

1 $$\mathrm{CO}_2 + \mathrm{H}_2 \leftrightarrow \mathrm{CO} + \mathrm{H}_2\mathrm{O}$$

and CH_3_OH in an exothermic methanol forming reaction (Eq. 2):

2 $$\mathrm{CO}_2 + 3\mathrm{H}_2 \leftrightarrow \mathrm{CH}_3\mathrm{OH} + \mathrm{H}_2\mathrm{O}$$

in both cases with the desorption of co-product H_2_O.

The rate and selectivity of these two reactions, which proceed simultaneously and by different pathways, facilitated by the SFLP, can be tailored advantageously by engineering the properties of the SFLP. In practice, this requires chemical means of adjusting the geometry of the SFLP and the negative and positive charge on the Lewis base and Lewis acid sites, respectively (Fig. 4a).

Note that these approaches to engineering the Lewis acidity and basicity of the SFLP refer to the electronic ground state of In_2_O_3-x_(OH)_y_. In the photo-excited state of In_2_O_3-x_(OH)_y_, the In2 and In1OH sites of the InOH•••In SFLP serve as traps for electrons and holes, respectively, making the proton and hydride of the SFLP H_2_ heterolysis intermediate InOH_2_^+^•••InH^−^ more acidic and basic towards subsequent reactions with CO_2_^32^. These traps also serve to lengthen the electron and hole excited state lifetimes, thereby enhancing the probability of reactions of InOH_2_^+^•••InH^−^ with CO_2_^33,34^. To this end, the charge-transfer dynamics of all samples were further investigated by time-resolved fluorescence spectroscopy (Supplementary Fig. 30). The curves can be fitted well using a triple exponential function (Supplementary Table 3 and Table 4). Following bandgap photoexcitation, electron-hole pairs relax into PL midgap SFLP defect sites, [O]/In(III) located closer in energy to the CB and OH similarly to the VB^33,34^. This non-radiative process into SFLP sites, likely corresponds to the shortest ns lifetime τ_3_. The electron-hole pair residing in the SFLP can relax radiatively to yield the observed defect PL or non-radiatively to the electronic ground state as phonons or chemically reacting with reactants CO_2_/H_2_ (Supplementary Fig. 31). Notably, rh-350 with the longest average PL lifetime has the superior photocatalytic activity. This is another way of engineering the activity and selectivity of the SFLP. The solar advantage stems from the greater excited state Lewis acid and Lewis basicity of the SFLP of In_2_O_3-x_(OH)_y_, which is manifest experimentally in lower activation energies for the excited state pathway compared with the ground state.

Another point worth mentioning is the adsorption strength of intermediates and products to the SFLP, which can influence selectivity and conversion rates of the CO and CH_3_OH pathways. For the case of a formate intermediate, created by the reaction of hydride with CO_2_, weak binding to the SFLP will favor the CO pathway (Supplementary Fig. 29, RWGS pathway), whereas strong binding will enable successive hydride transfers to formate, thereby favouring the CH_3_OH pathway (Supplementary Fig. 29, formate pathway). In addition, the strongly adsorbing, surface coordinating products H_2_O and CH_3_OH, which have to be desorbed to make the CO_2_ hydrogenation reactions catalytic, will act as rate limiting if the Lewis acidity of the In2 site is too high. Clearly, optimizing the rate and selectivity of CO_2_ hydrogenation to CO and CH_3_OH by In_2_O_3-x_(OH)_y_ requires a delicate balancing act of the Lewis acidity and Lewis basicity of the SFLP towards the binding of reactants, intermediates and products.

Approaches to SFLP engineering that have proven successful so far include control of oxygen vacancies and isomorphous substitution of the indium sites^6,29^. In the case of [O]_v_ vacancies, the higher the substituting population is, the lower the oxygen coordination number around the In(III) sites. This makes the In1OH more Lewis basic and the coordinately unsaturated In2 more Lewis acidic. For isomorphous substitution of In(III) by a similar-sized yet more electronegative element like Bi(III), exchange of the In2 site renders it more Lewis acidic, whereas replacement of In1 makes the hydroxide less Lewis basic.

Another strategy for tailoring the SFLP would be to change the distance between the Lewis acid and Lewis base sites (Fig. 4a), which would alter their charges and modify how they interact with H_2_. The geometry of the SFLP will vary between different crystal facets, which to implement will require strict control of the crystal morphology. Geometry changes of the SFLP are also achievable by polymorph engineering, a more straightforward method in practice, and the subject of the experimental and computational studies described herein.

In summary, we have demonstrated a polymorph selection strategy to modify the Lewis acidity and Lewis basicity of rhombohedral In_2_O_3-x_(OH)_y_ with a view to tuning activity and selectivity in gas-phase CO_2_ (photo)catalysis. Significantly, rh-In_2_O_3-x_(OH)_y_ turns out to be a high performance photocatalyst, achieving champion CO_2_ hydrogenation rates to CH_3_OH and CO at atmospheric pressure. The superior catalytic performance appears to originate in the enhanced activity of surface Lewis acid–base pairs and strong propensity towards H_2_ dissociation. An operando DRIFT study and DFT calculation provide information on the surface chemistry responsible for the formation of CH_3_OH and CO, which appear to proceed by different reaction pathways. Based on the results and insight gained from this work, it should prove possible to optimize the Lewis acidity and Lewis basicity and enhance the photocatalytic performance of heterogeneous SFLP photocatalysts through polymorph selection. Furthermore, by understanding the distinct light-dependence of CO and CH_3_OH formation and the impact of the electronic structure on CO_2_ activation and H_2_ dissociation by cubic and rhombohedral In_2_O_3-x_(OH)_y_, these heterogeneous SFLP systems can be incorporated into multi-component catalytic systems exemplified by polymorphic heterostructures, with distinct structures yet continuously adjustable fractions, enabling efficient CO_2_ hydrogenation with front-line status.

## Methods

### Chemicals

All reagents used in the present study, including *N*,*N*-dimethylformamide (DMF), Indium(III) nitrate hydrate (In(NO_3_)_3_·4.5H_2_O, In 29%), and ethanol (C_2_H_5_OH) were analytical reagent grade and obtained from Sigma-Aldrich. All chemicals were used as received. Deionized water was used throughout the synthesis.

### Synthesis of InOOH precursor and rhombohedral In_2_O_3-x_(OH)_y_ nanocrystals

In a typical synthesis of InOOH precursor, 0.3 g of In(NO_3_)_3_·4.5H_2_O and 0.8 mL of distilled water were added to a 25 mL autoclave. DMF was then added to bring the total volume up to 17 mL. The aqueous solution was then heated at 150 °C for 24 h. After being cooled to room temperature, the white products were collected through centrifugation and washed with water and ethanol. The sample was finally dried at 60 °C. The dried InOOH precursors were then placed into an oven and treated at various temperatures (250–400 °C) in air for 4 h to obtain the final In_2_O_3-x_(OH)_y_ samples.

### Characterization

PXRD was performed on a Bruker D2-Phaser X-ray diffractometer, using Cu Ka radiation at 30 kV. The HRTEM measurement was conducted using a JEM–2010 microscope working at 200 kV. Nitrogen Brunauer–Emmet–Teller (BET) adsorption isotherms were obtained using an ASAP2020 M apparatus (Micromeritics Instrument Corp., USA). For BET surface area analyses, the samples were degassed in vacuum at 110 °C for 10 h and then measured at 77 K. The weight loss of InOOH precursor was carried out in a TA Instruments SDT Q600 thermogravimetric analyzer in an alumina pan under 100 mL min^−1^ flow of compressed air. The temperature was steadily increased from room temperature (25 °C) to 580 °C at a rate of 5 °C min^−1^. UV-visible diffuse reflectance spectra of the powders were obtained for the dry-pressed disk samples using a Cary 500 Scan Spectrophotometer (Varian, USA) over a range of 200–800 nm. BaSO_4_ was used as a reflectance standard in the UV-visible diffuse reflectance experiment. XPS was performed using a PerkinElmer Phi 5500 ESCA spectrometer in an ultrahigh vacuum chamber with base pressure of 1 × 10^−9^ Torr. The spectrometer uses an Al Ka X-ray source operating at 15 kV and 27 A. The samples were coated onto carbon tape, and all results were calibrated to C1s 284.5 eV. The room temperature photoluminescence (PL) spectrum was measured on a FL/FS 920 (Edinburgh Instruments) equipped with a 450 W Xe arc lamp as the excitation source and a red sensitive Peltier element cooled Hamamatsu R2658 PMT as the detector. Time-resolved fluorescence decay spectra were recorded on the Delta Pro (HORIBA instruments) using a 357 nm laser as the excitation source. Carbon dioxide temperature-programmed desorption (CO_2_-TPD) measurements were performed on a Micromeritics AutoChem II 2920 chemisorption analyzer.

### Gas-phase photocatalytic measurements

The gas-phase CO_2_ hydrogenation experiments were conducted in an inner diameter of 2 mm tubular quartz reactor, in which ∼ 20 mg of catalyst sample was packed into and fully irradiated with an unfiltered 130 W Xe lamp. The diameter of the light spot was ~ 2 cm, with an area of about 3.14 cm^2^, which could fully cover the sample. An OMEGA temperature controller was attached to a heating cartridge inserted into the copper block along with a thermocouple inserted into the quartz tube in contact with the catalyst bed for control of the catalyst temperature. In a typical run, CO_2_ or ^13^C isotope-labeled CO_2_ (99 atom% 13 C; Sigma) and H_2_ with a ratio of 1: 3 (2 mL min^−1^ and 6 mL min^−1^) were introduced into the reactor by Alicat Scientific digital flow controllers. The amounts of CO and CH_3_OH produced were analyzed by an on-line gas chromatograph (Agilent 7820 A), equipped with a thermal conductivity detector (TCD) and a flame ionization detector (FID).

### In situ DRIFTS measurements

In situ DRIFTS measurements were performed to detect and characterize the possible surface intermediates over rhombohedral phase of In_2_O_3-x_(OH)_y_ nanocrystals under reaction conditions. The spectra were collected using a Fourier-transform infrared spectroscopy spectrometer (Thermo, Nicolet 6700) equipped with an MCT detector. Before measurement, the catalyst was purged with He at 350 °C for 2 h. The catalyst was subsequently cooled down to 230 °C. The background spectrum with a resolution of 4 cm^−1^ was obtained at 230 °C in He flow. Then the catalyst was exposed to a CO_2_/H_2_/He mixture (1 mL min^−1^ CO_2_, 3 mL min^−1^ H_2_ and 16 mL min^−1^ He) for 30 min. The in situ DRIFT spectra were recorded by collecting 32 scans at 4 cm^−1^ resolution.

### DFT calculations

Theoretical calculations are carried out with the context of DFT, as implemented in the Vienna ab initio simulation package. The exchange–correlation interactions were treated within the generalized gradient approximation in the form of the Perdew−Burke−Ernzerhof functional. The projector augmented wave approach with plane wave cutoff energy of 400 eV is used, and the convergence criteria are set to be 10^−4^ in energy and 0.02 eV Å^−1^ in force. To model the rhombohedral In_2_O_3_ surfaces, four-layer slabs within vacuum spacing larger than 20 Å with the bottom two layers keep fixed are adopted. Brillouin zone integrations are performed over the Gamma point owing to the large supercell. Nudged elastic band method is used to determine the H_2_ dissociation path and barrier.

## Supplementary information

Supplementary Information

## Data Availability

The data that support the plots within this paper and other findings of this study are available from the corresponding author upon reasonable request.
