# Supplementary material for: Polymorph selection towards photocatalytic gaseous CO2 hydrogenation
Source: Nat Commun. 2019 Jun 7;10:2521. doi: 10.1038/s41467-019-10524-2 (PMC6555785; doi:10.1038/s41467-019-10524-2)
Supplement: Supplementary file 1 — Supplementary Information [file 41467_2019_10524_MOESM1_ESM.pdf]

Supplementary Information

Polymorph Selection Towards Photocatalytic Gaseous CO_2 Hydrogenation

Yan *et al.*

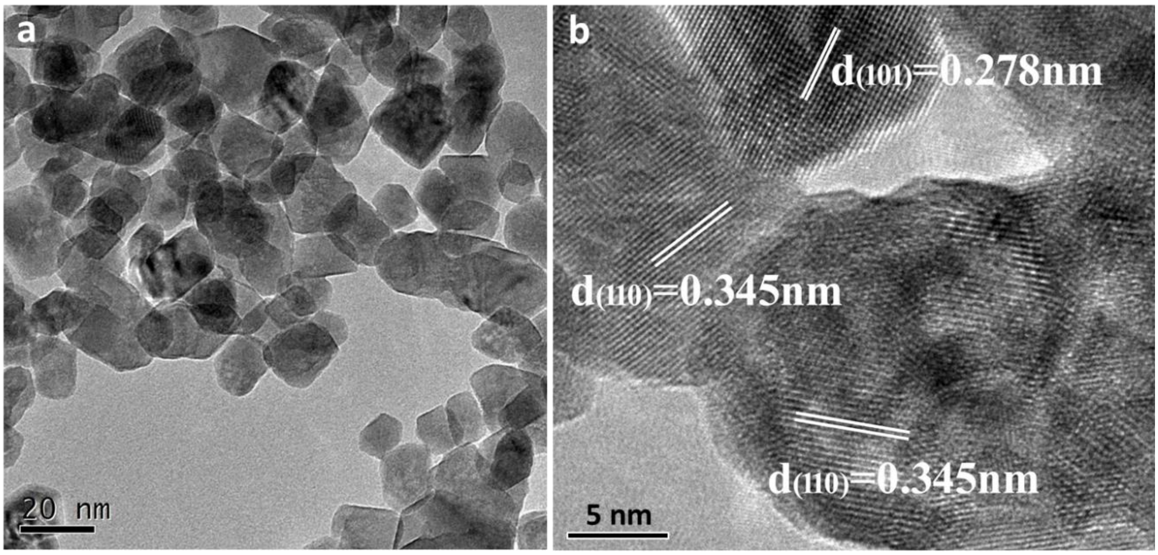

Supplementary Figure 1. (a) TEM image and (b) HRTEM image of InOOH precursor.

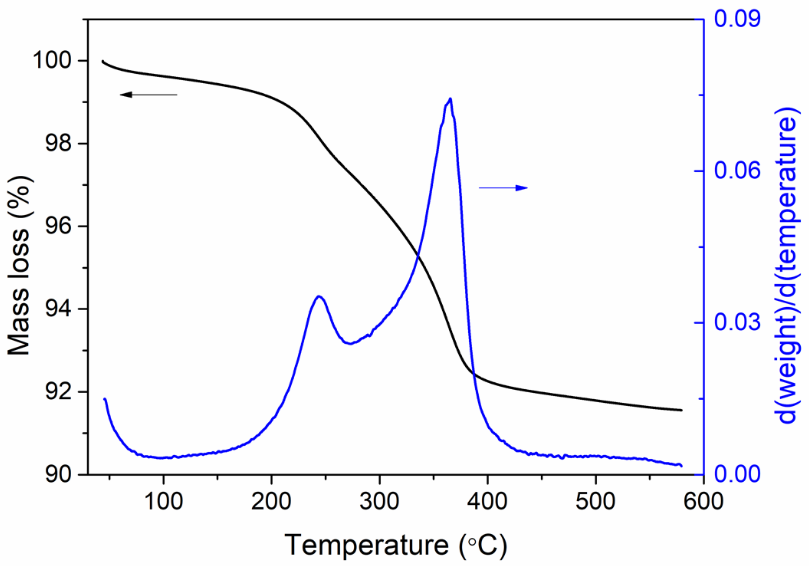

Supplementary Figure 2. TG and DTG curves of InOOH precursor tested in air at temperature raising rate of 5 °C min^{-1}. The dehydroxylation and phase conversion of the InOOH precursor was tested using thermogravimetric (TG) analysis and differential thermogravimetric (DTG) analysis. The curve can be divided into three weight loss steps. The first step occurs at 50-180 °C and accounts for about 1.68 % of weight loss, which corresponds to the physical water evaporation. The second step between 180-270 °C along with an endothermic peak at ~244 °C can be attibuted to the removal of residual DMF. A sharp weight loss is observed in the third step from 270 to 450 °C, accompanied by an endothermic peak at ~366 °C, can be considered as the conversion of InOOH into $In_2O_{3-x}(OH)_y$ through dehydroxylation of the hydroxyl groups. The total weight loss for this step is 6.05 %, which is very close to the theortical loss (6.08 %) calculated by the reaction $2InOOH \rightarrow In_2O_3 + H_2O$.

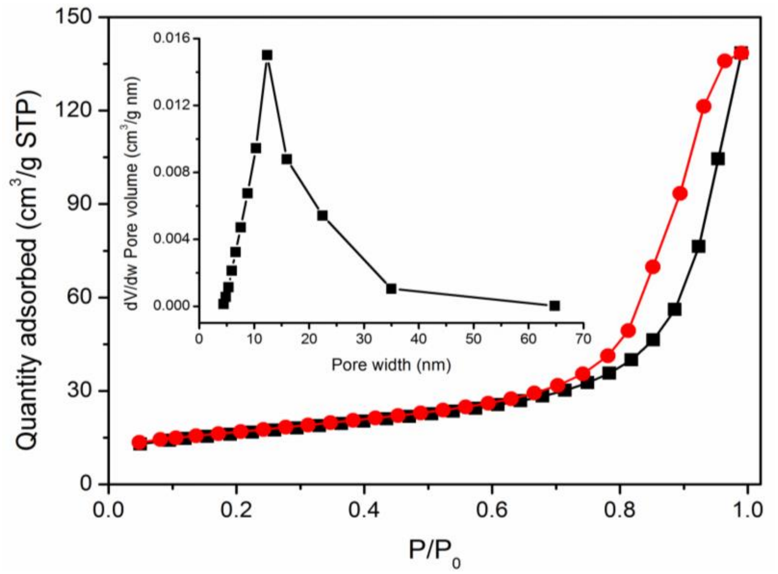

Supplementary Figure 3. N_2 physisorption isotherms (adsorption black, desorption red branches) and pore size distributions of rh-$In_2O_{3-x}(OH)_y$ nanocrystals.

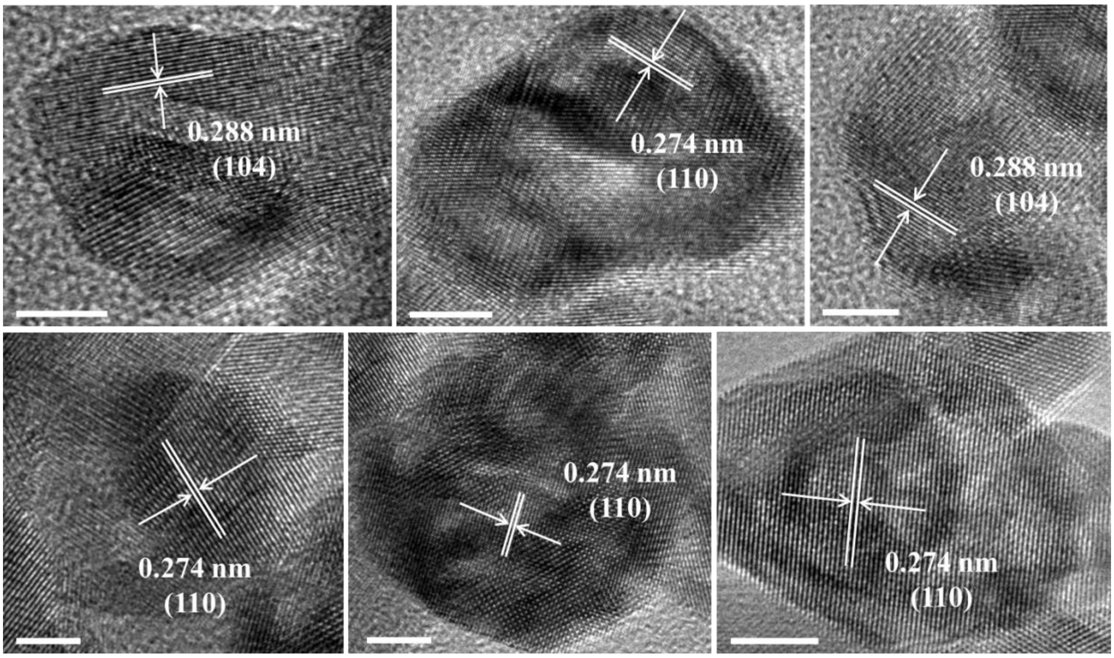

Supplementary Figure 4. HRTEM images of rh-$In_2O_{3-x}(OH)_y$ nanocrystals. Scale bar, 5 nm.

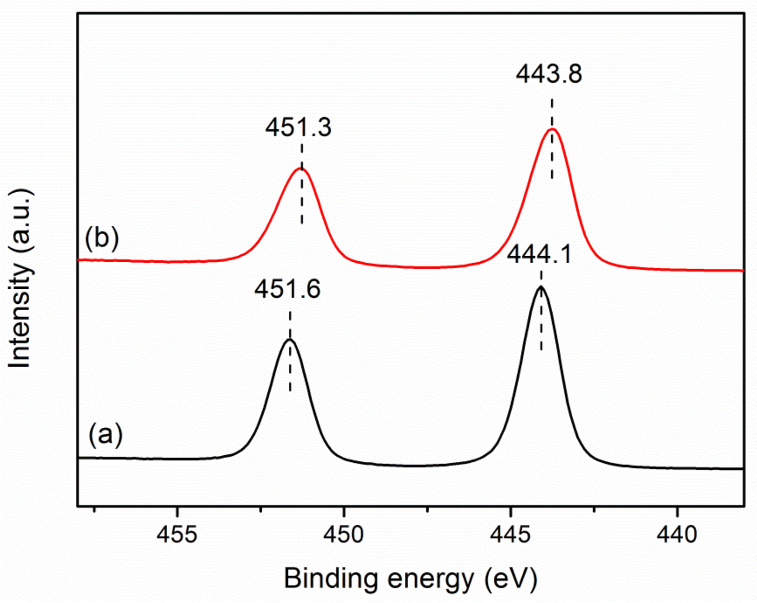

Supplementary Figure 5. XPS spectra of In3d for (a) InOOH precursor and (b) rh-$In_2O_{3-x}(OH)_y$ nanocrystals.

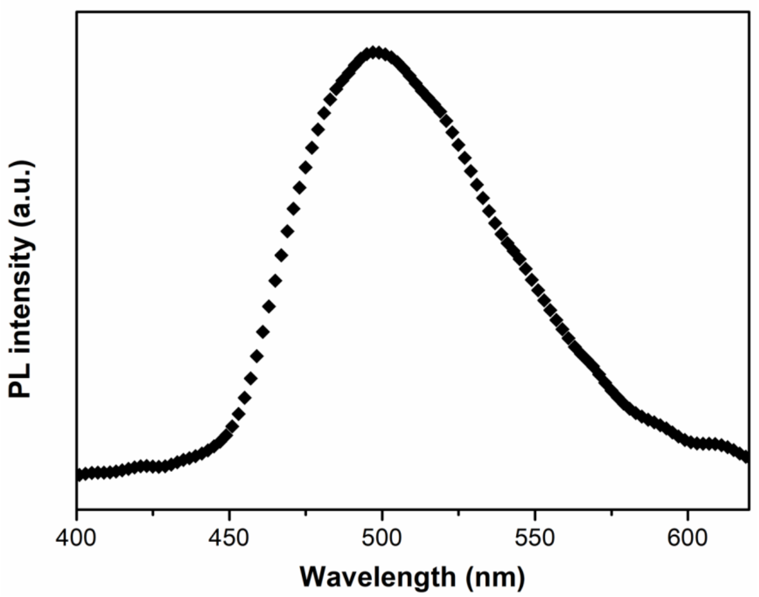

Supplementary Figure 6. Room-temperature PL spectrum of rh-$In_2O_{3-x}(OH)_y$ nanocrystals using an excitation wavelength of 325 nm.

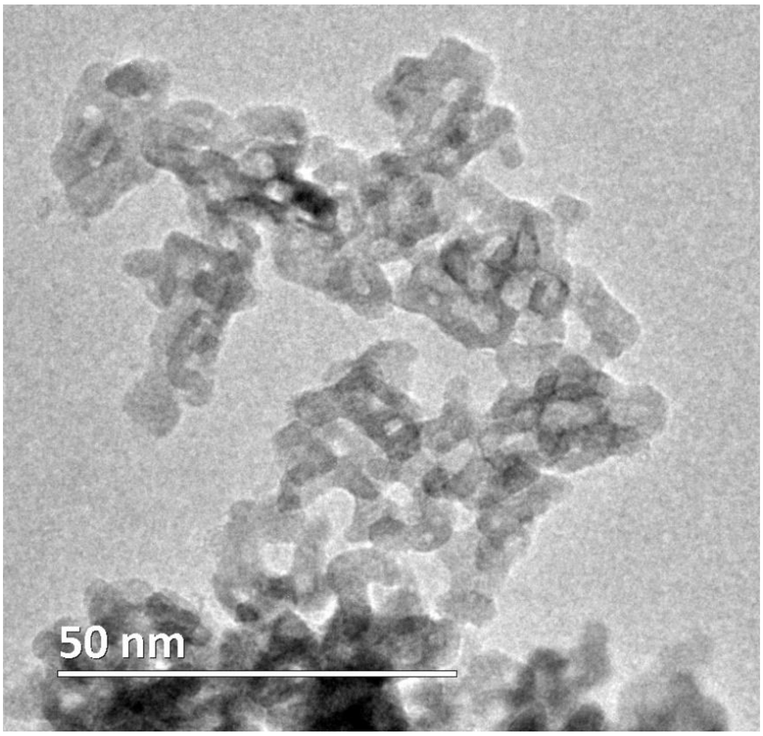

Supplementary Figure 7. TEM image of c-$In_2O_{3-x}(OH)_y$ nanocrystals.

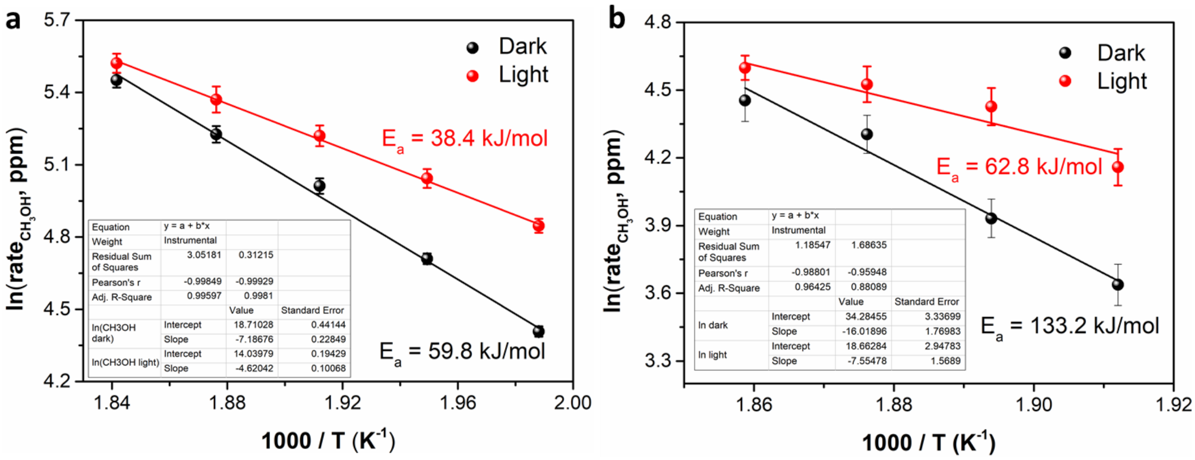

Supplementary Figure 8. The Arrhenius plots for the production rate of CH_3OH on (a) rh-$In_2O_{3-x}(OH)_y$ and (b) c-$In_2O_{3-x}(OH)_y$ recorded at different reaction temperatures with and without light irradiation.

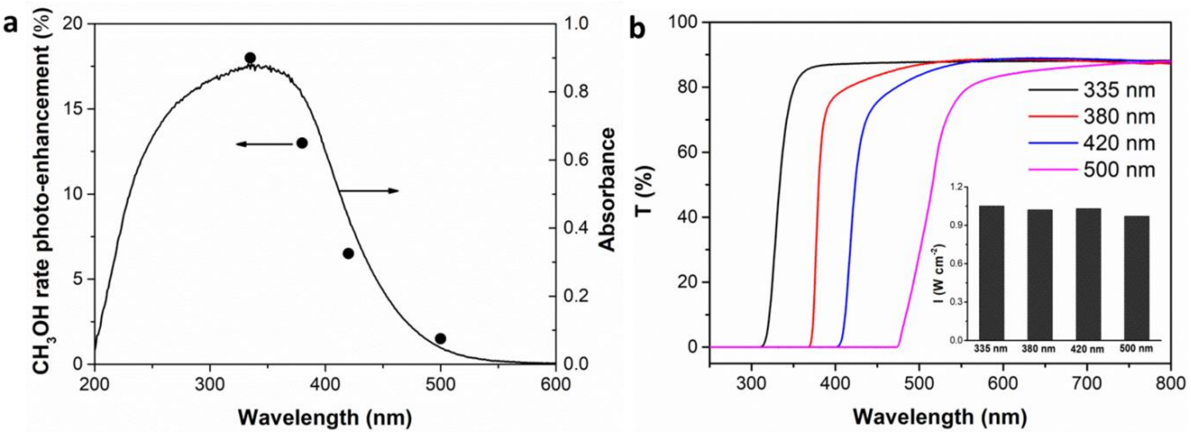

Supplementary Figure 9. (a) Wavelength dependence of the photocatalytic CH_3OH rate enhancement on rh-$In_2O_{3-x}(OH)_y$ upon irradiation with cut-off filters of different wavelengths (with respect to thermal condition at 270 °C), and (b) transmittance of the used light filters. Inset: wavelength dependence of light intensity (W cm^{-2}) with different cut-off filters.

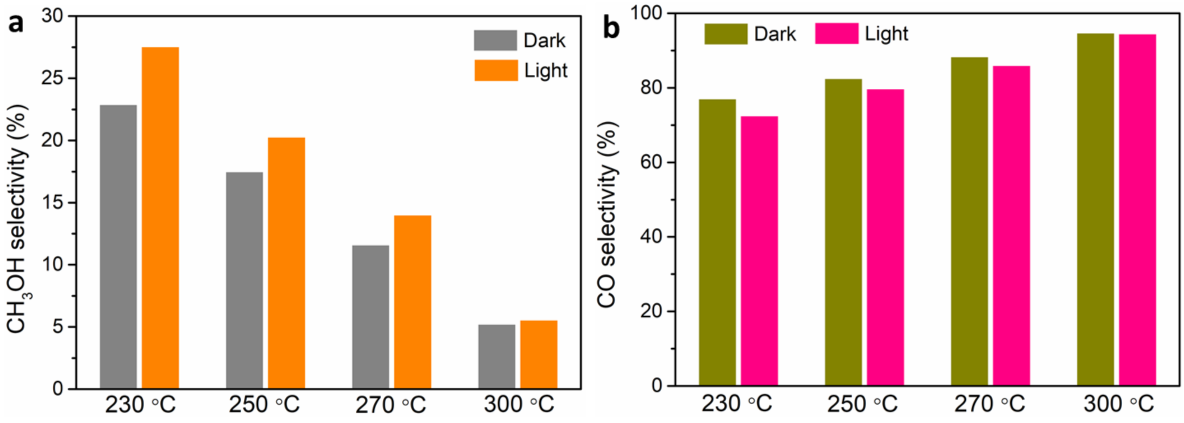

Supplementary Figure 10. (a) CH_3OH and (b) CO selectivity at different reaction temperatures with and without solar irradiation on rh-$In_2O_{3-x}(OH)_y$.

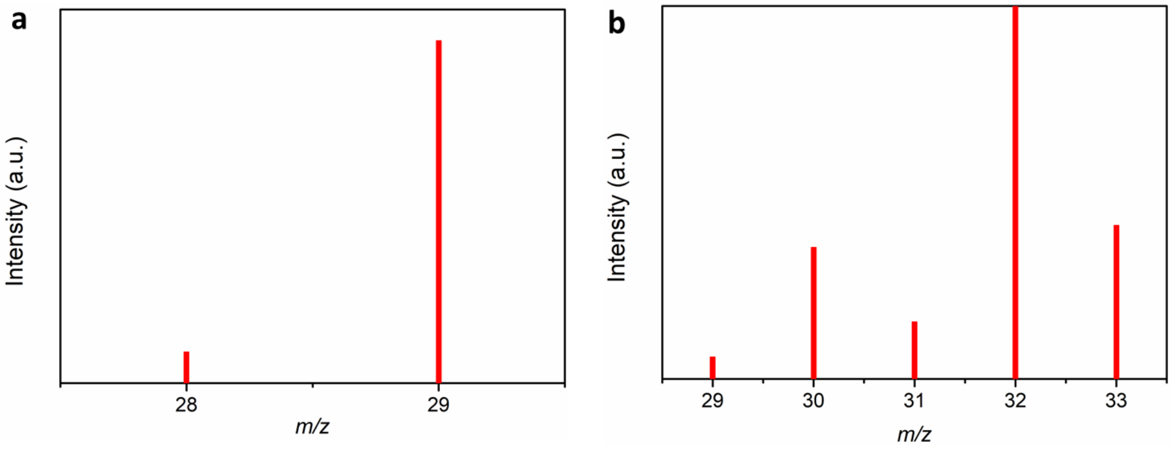

Supplementary Figure 11. Isotope-labelled mass spectrum of (a) CO and (b) CH_3OH products using $^{13}CO_2$ feedstock.

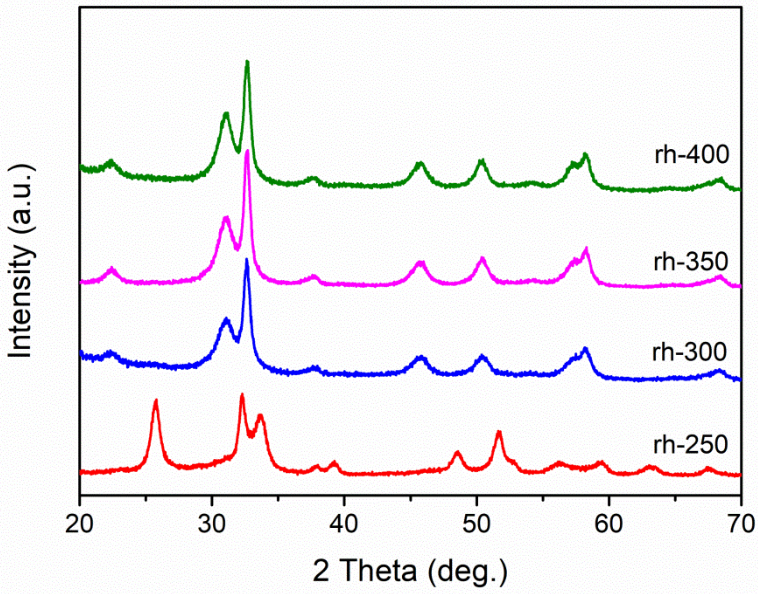

Supplementary Figure 12. XRD patterns of $In_2O_{3-x}(OH)_y$ nanocrystals annealed at different temperatures.

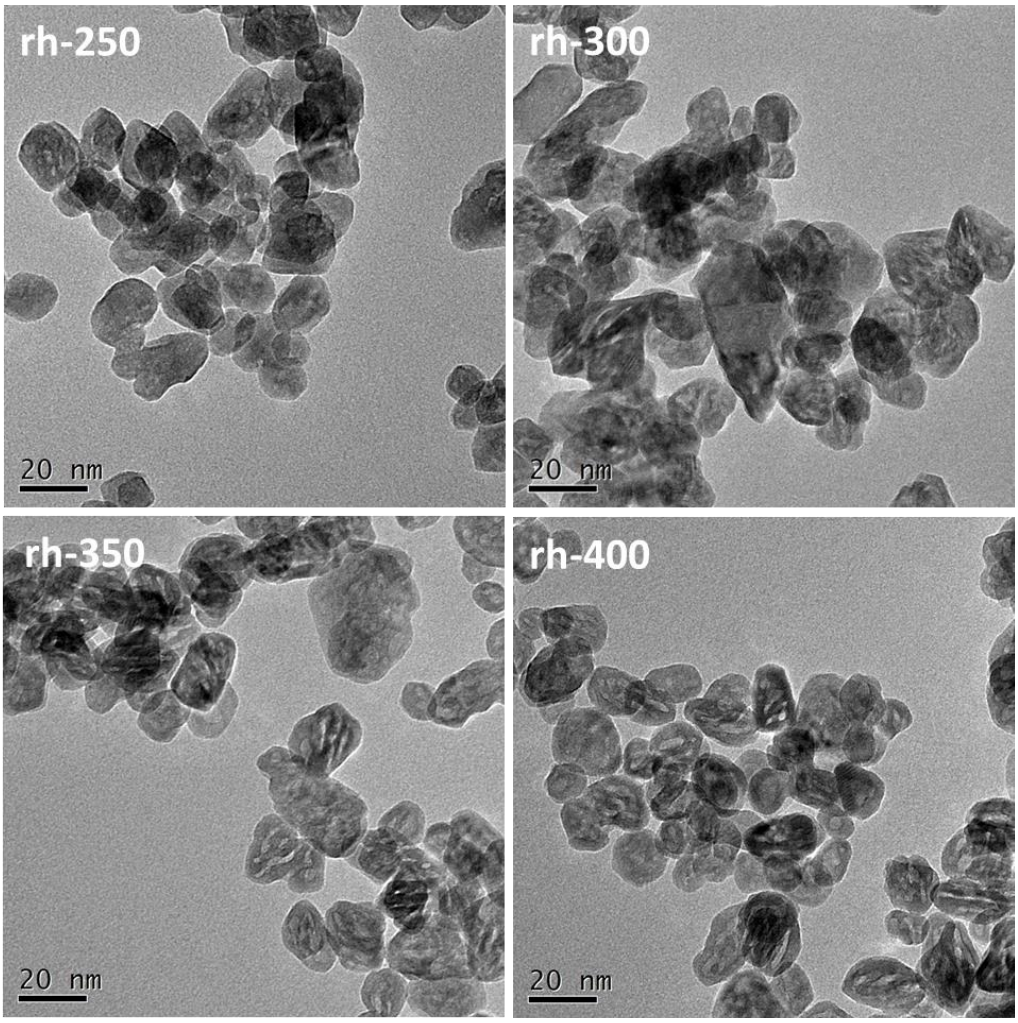

Supplementary Figure 13. TEM images of various $In_2O_{3-x}(OH)_y$ nanocrystals annealed at different temperatures.

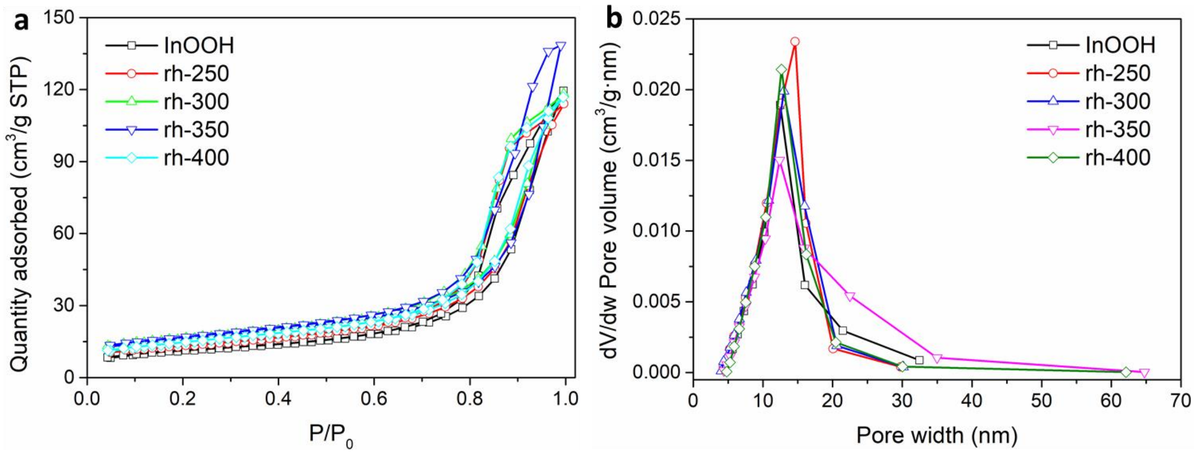

Supplementary Figure 14. (a) N_2 physisorption isotherms (adsorption-desorption branches), and (b) pore size distributions of the InOOH precursor and $In_2O_{3-x}(OH)_y$ nanocrystals formed by thermal treatment of the inOOH precursor at the temperatures indicated.

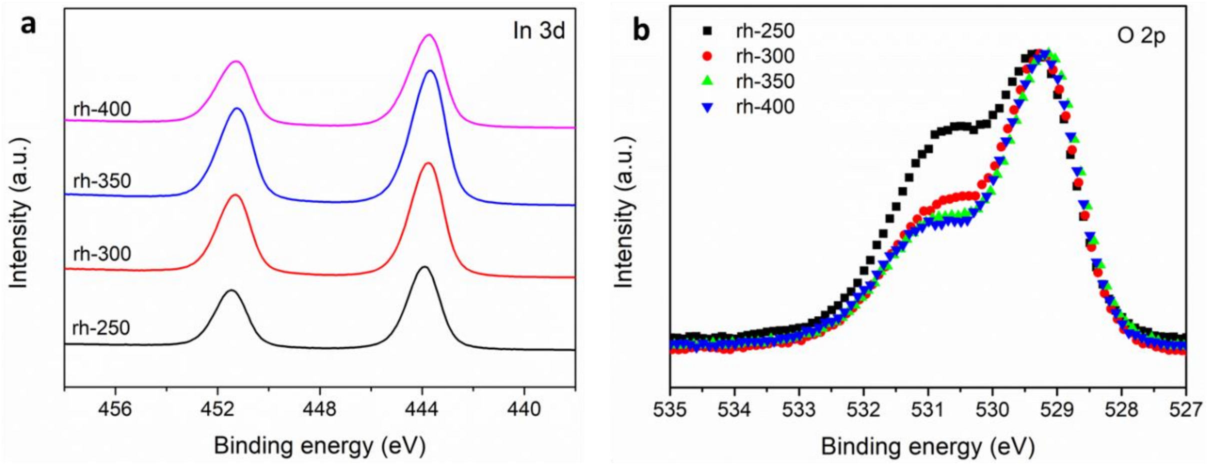

Supplementary Figure 15. (a) In 3d XPS spectra and (b) O 1s XPS spectra for $In_2O_{3-x}(OH)_y$ nanocrystals formed by thermal treatment of the inOOH precursor at the temperatures indicated.

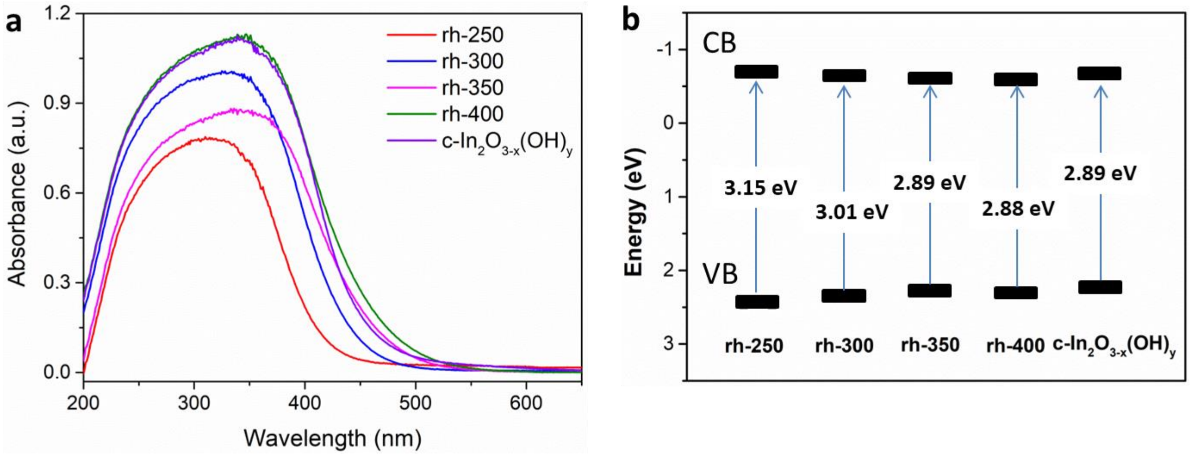

Supplementary Figure 16. (a) Diffuse reflectance spectra (DRS) and (b) electronic band structures of rhombohedral and cubic $In_2O_{3-x}(OH)_y$ nanocrystals. The conduction band (CB) was measured by Mott-Schottky experiments (not shown) and the band gap was calculated by fitting the reflectance spectra using K-M theory.

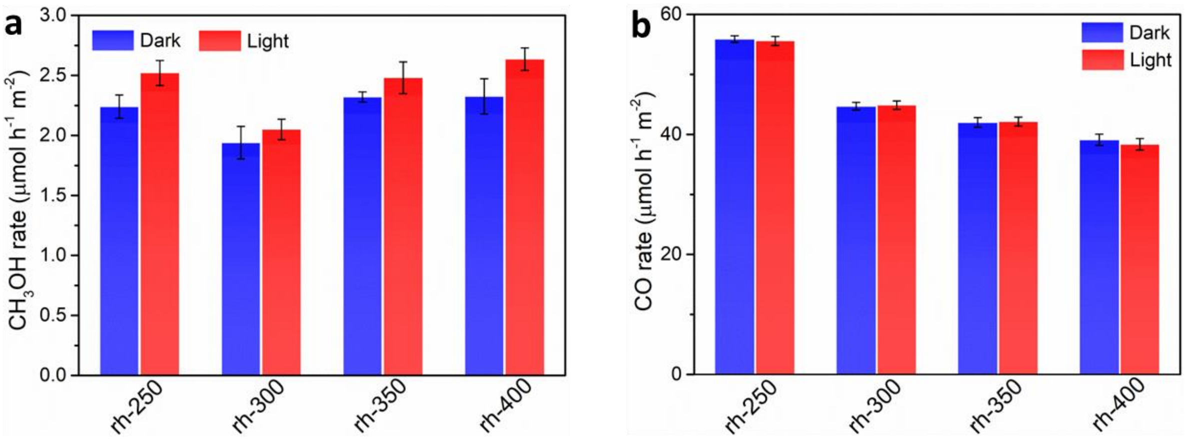

Supplementary Figure 17. (a) Surface area normalized CH_3OH production rate and (b) Surface area normalized CO production rate at 300°C with and without solar irradiation on various $In_2O_{3-x}(OH)_y$ nanocrystals.

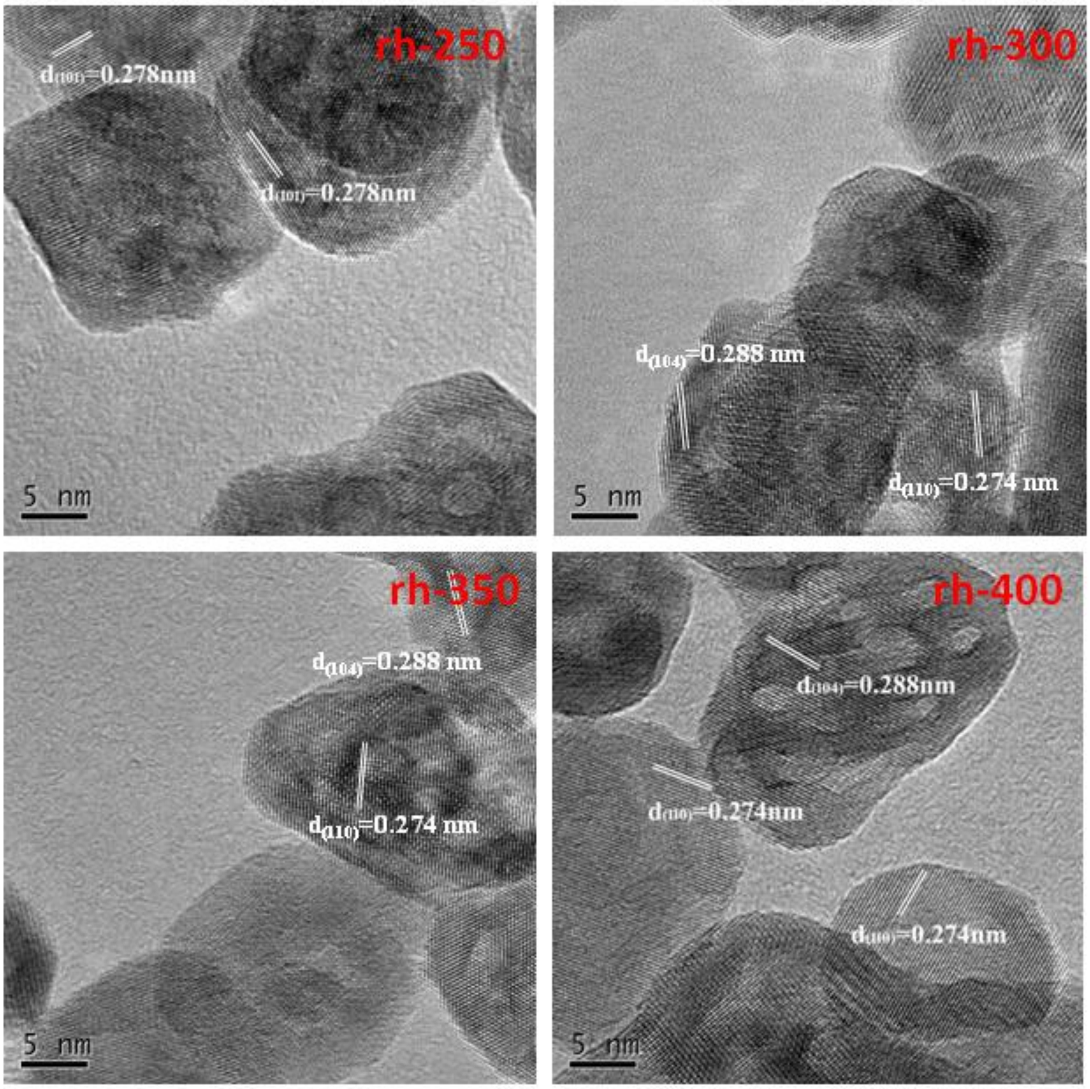

Supplementary Figure 18. HRTEM images of various $In_2O_{3-x}(OH)_y$ nanocrystals.

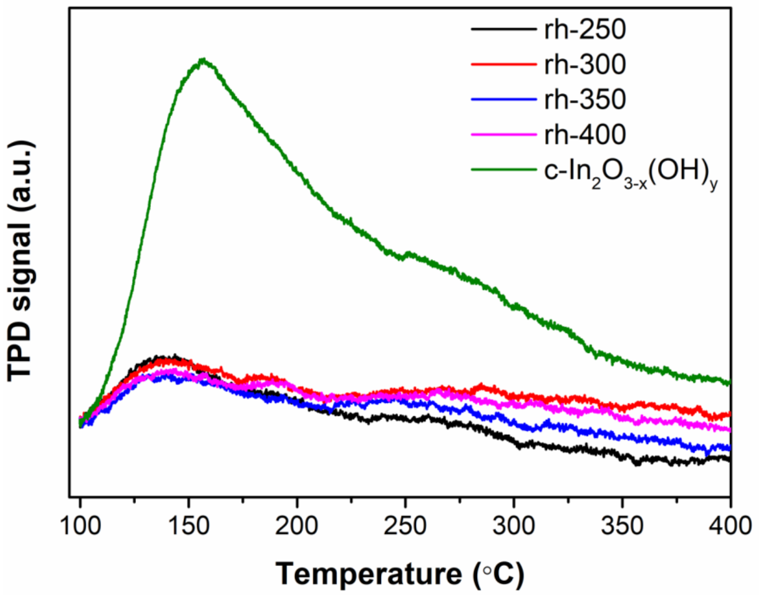

Supplementary Figure 19. CO_2-TPD profiles of various $In_2O_{3-x}(OH)_y$ nanocrystals.

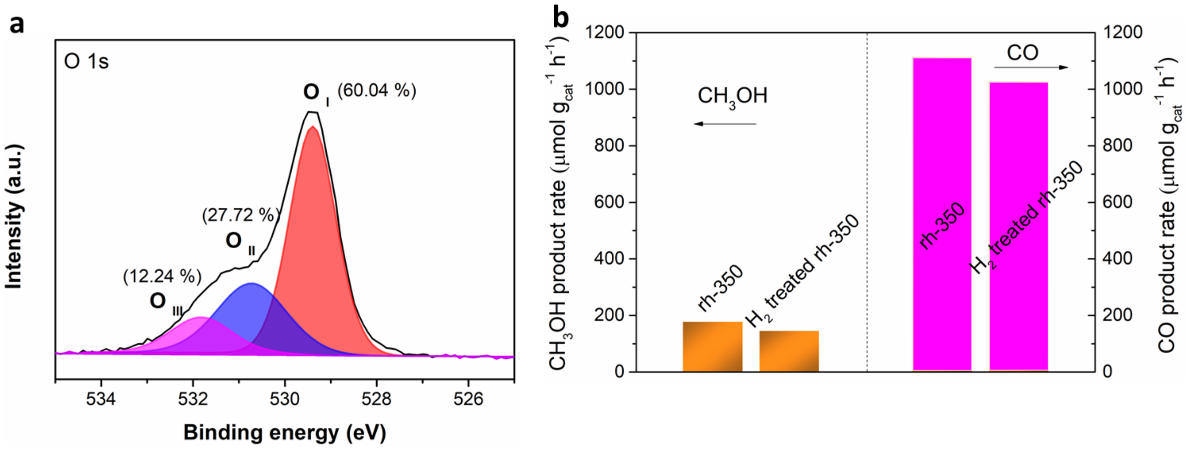

Supplementary Figure 20. (a) O 1s XPS spectrum of rh-350 after H_2 treatment, and (b) the CH_3OH and CO production rates at 270°C with solar irradiation on the rh-350 and H_2 treated catalyst. To treat the rh-350 catalyst, the sample was placed into a tubular furnace in a 5% H_2/He flow for 1 hour at 270 °C.

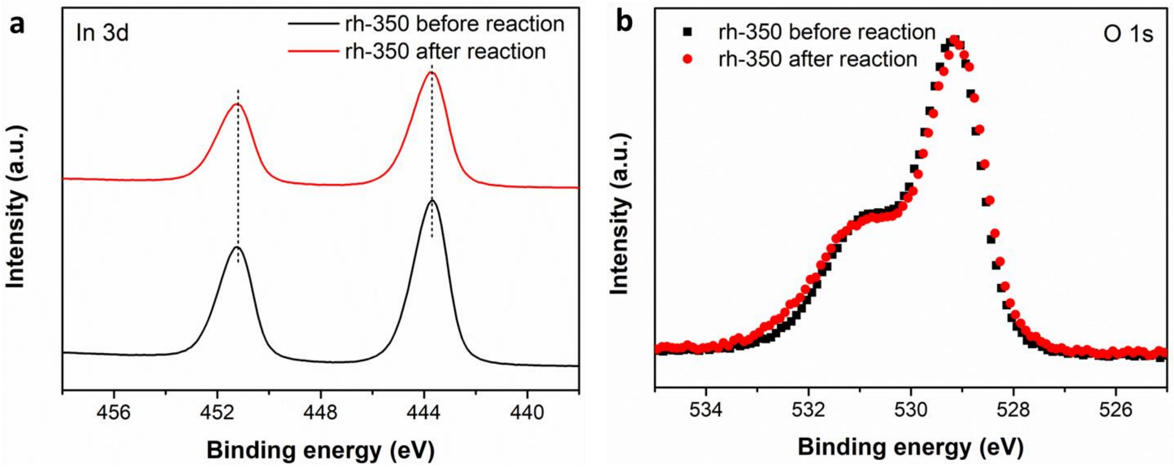

Supplementary Figure 21. (a) In 3d and (b) O1s XPS spectra of $In_2O_{3-x}(OH)_y$ nanocrystals (rh-350) before and after the 100 h long-term catalyst stability test.

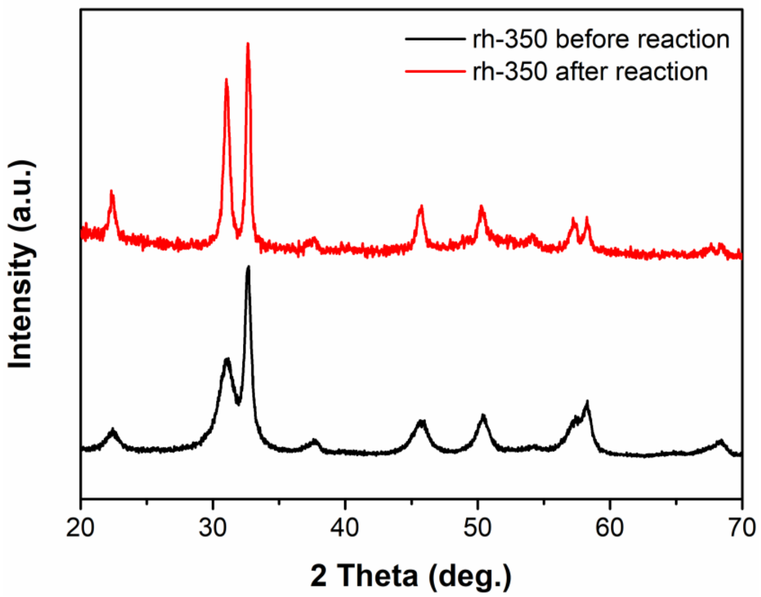

Supplementary Figure 22. PXRD patterns of the $In_2O_{3-x}(OH)_y$ nanocrystals (rh-350) before and after the 100 h long-term catalyst stability test.

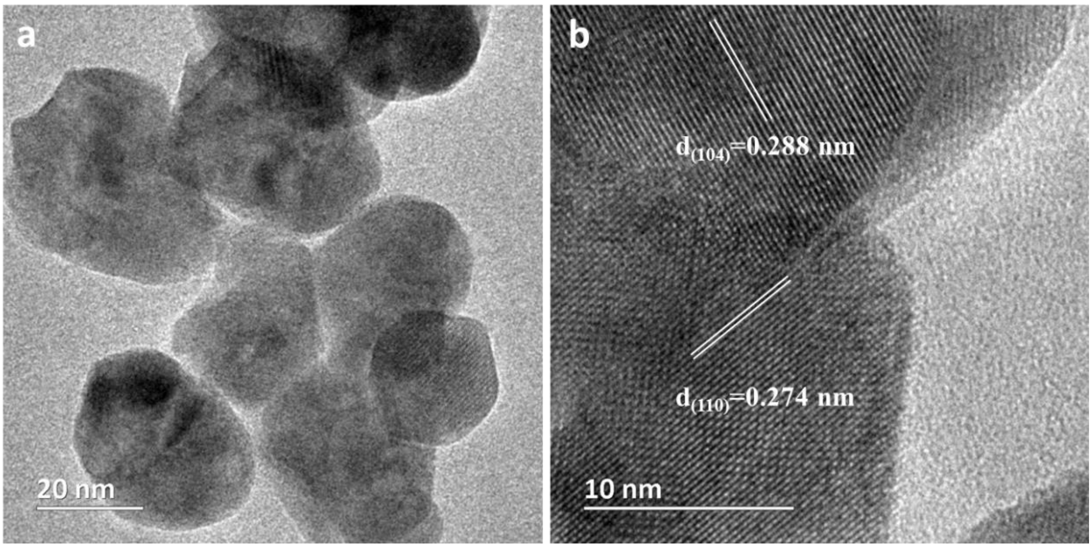

Supplementary Figure 23. (a) TEM and (b) HRTEM images of $In_2O_{3-x}(OH)_y$ nanocrystals (rh-350) after the 100 h long-term catalyst stability test.

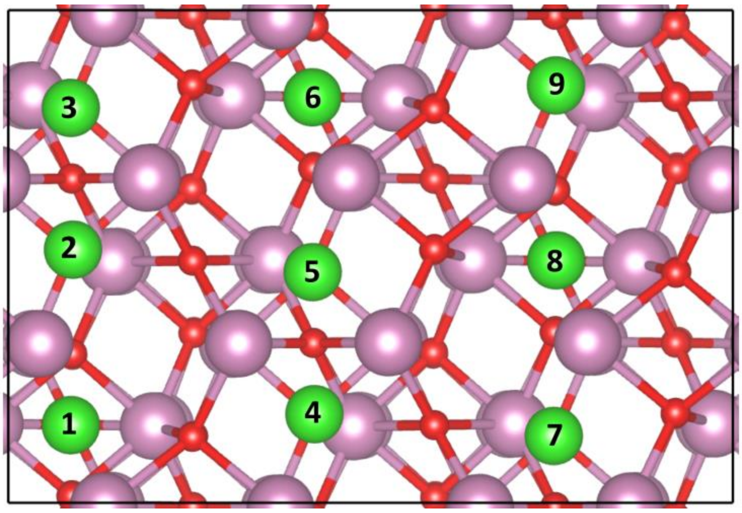

Supplementary Figure 24. Surface vacancy sites of rhombohedral $In_2O_{3-x}OH_y$ nanostructure at (110) direction. The color-coding contains pink, red, and green for In, O, and O vacancy; respectively.

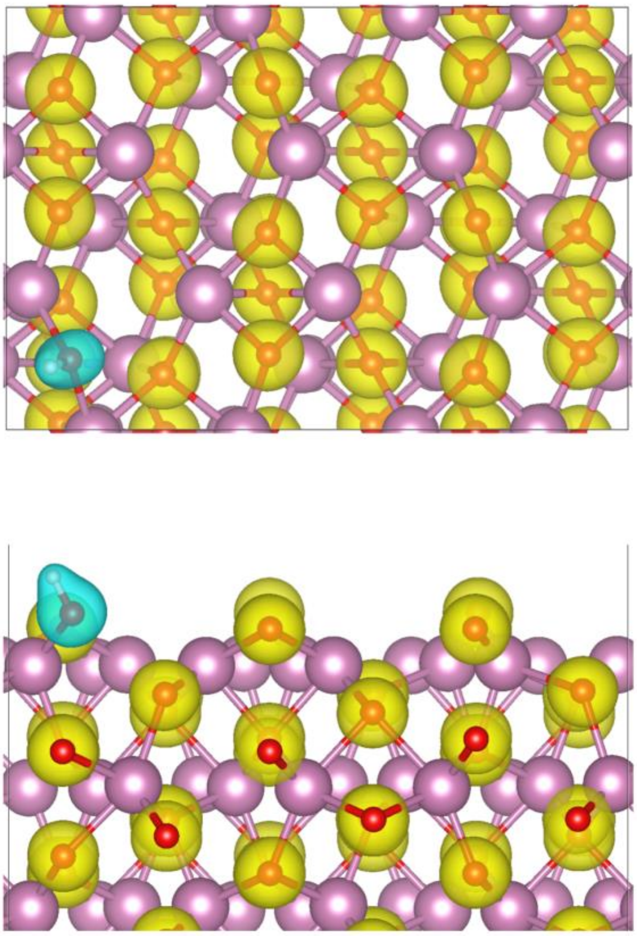

Supplementary Figure 25. Top and side views for the (110) rhombohedral $In_2O_{3-x}(OH)_y$ surface. The color-coding contains pink, red, and white for In, O, and H, respectively.

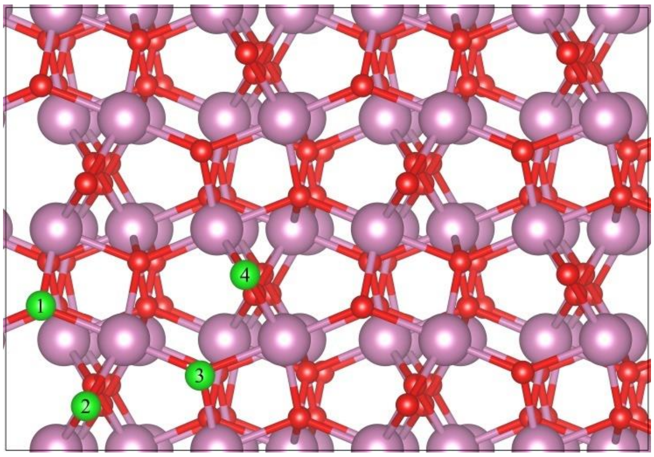

Supplementary Figure 26. Top view of surface structure of (104) terminated rhombohedral $In_2O_{3-x}OH_y$ with possible oxygen vacancy sites (green spheres). DFT calculations are also conducted for the (104) surface of rh-$In_2O_{3-x}OH_y$ crystal in the present study. Four possible oxygen vacancy sites labelled as 1 to 4 are presented. The theoretical calculations indicate that the surface vacancy at site 4 is most energrtically favorable with formation energy of 1.86 eV.

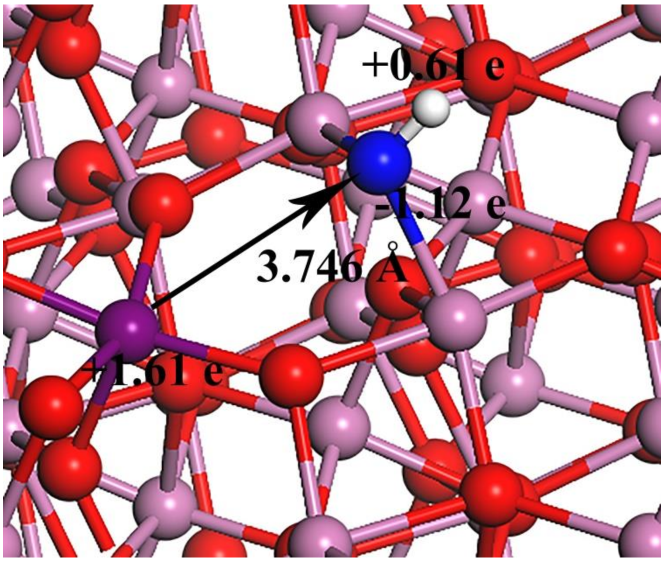

Supplementary Figure 27. Optimized configuration for (104) rhombohedral $In_2O_{3-x}(OH)_y$ surface. After the OH group binds at the vacancy site 4, the corresponding Bader charges are depicted. It reveals that 0.39e transfer from H atom to O in OH group, the Lewis acidic In and Lewis basic O of the OH sites at the surface possess charges of +1.61e and -1.12e, respectively. Additionally, the distance between Lewis acid and base is 3.746 Å. These factors also likely contribute to the activation of small molecules akin to (111) surface of c-$In_2O_{3-x}(OH)_y$.

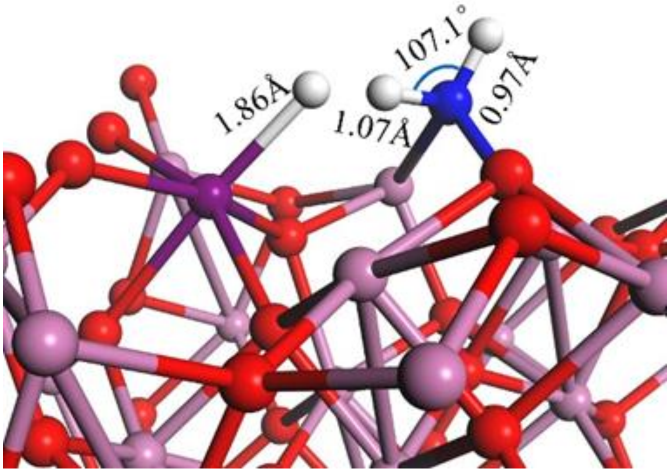

Supplementary Figure 28. Optimized configuration for hydrogenated (110) rhombohedral $In_2O_{3-x}(OH)_y$. Due to the strong electric field induced by Lewis acid–base pairs in (110) rh-$In_2O_{3-x}(OH)_y$, H_2 is readily polarized (evident from Bader charges of +0.34e and -0.56e, respectively) and ultimately bind to OH and In. The H-H distance of H_2 is enlarged from 0.75 Å to 1.24 Å and the newly formed In-H and H-OH bonds are 1.86 and 1.07 Å, respectively. The bond angle of the H-OH structure is 107.1°.

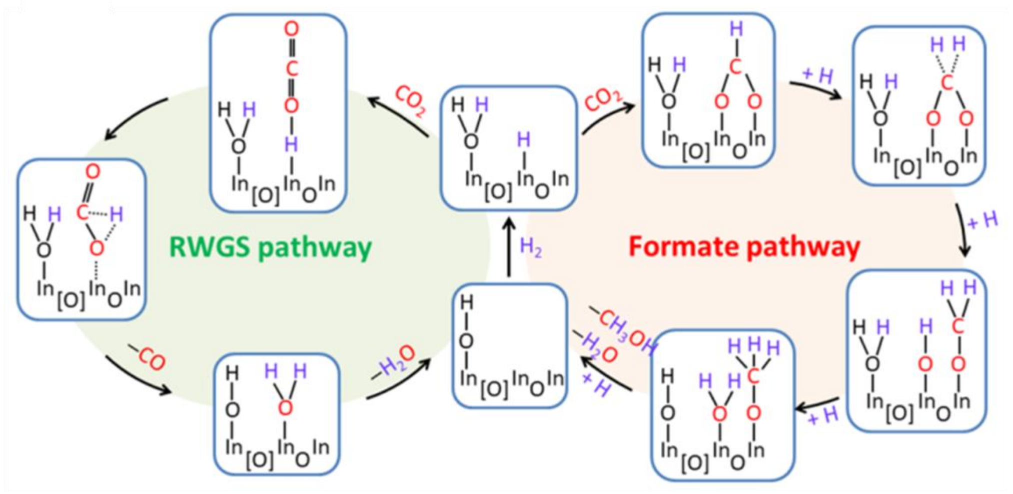

Supplementary Figure 29. Reaction scheme for CO_2 hydrogenation to CO and CH_3OH via the RWGS and formate pathways.

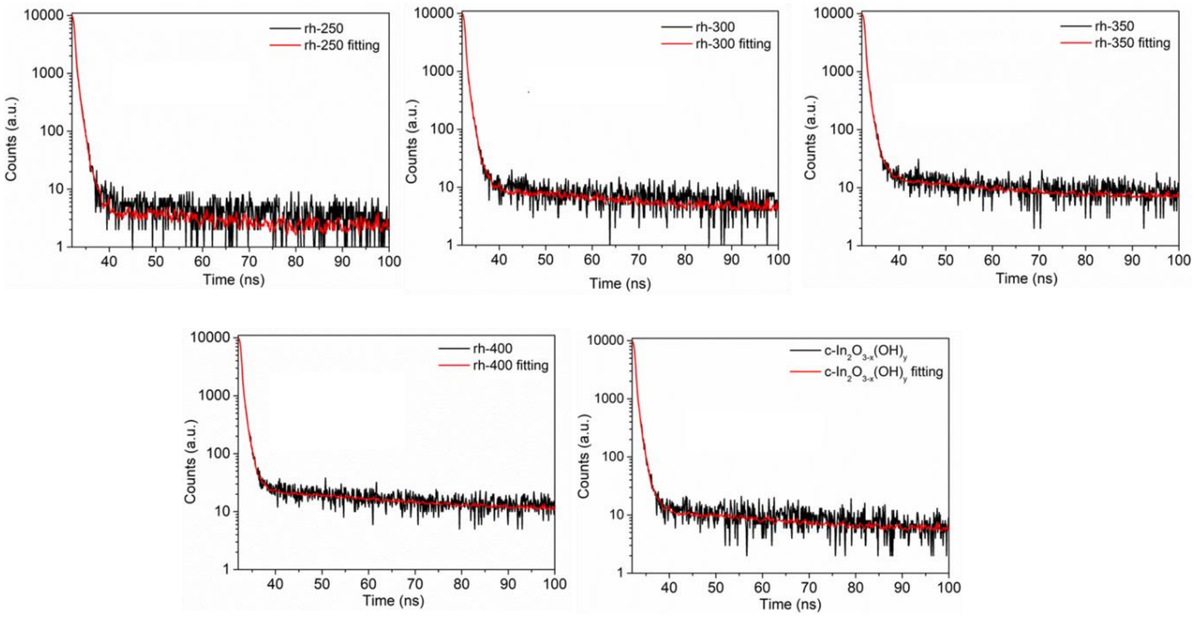

Supplementary Figure 30. The time-resolved fluorescence decay profiles of various $In_2O_{3-x}(OH)_y$. The fluorescence lifetimes were determined from the decay curves of the emission centered at ca. 500 nm, as shown in Supplementary Figure 6. The average lifetimes (τ) were calculated by fitting the curves with a triple exponential function (Supplementary Table 3), which provides a better fit than a double exponential function (Supplementary Table 4).

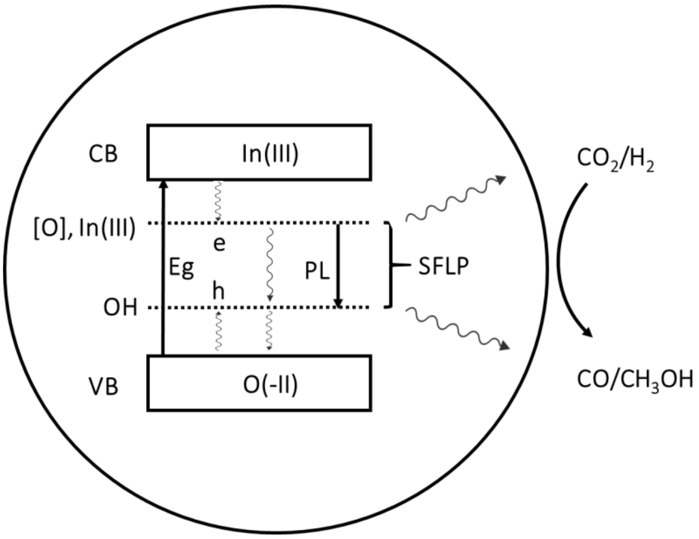

Supplementary Figure 31. Schematic illustration of charge carrier recombination pathways in rh-$In_2O_{3-x}(OH)_y$ nanoparticles. The solid black arrows represent bandgap absorption Eg and defect PL emission, the wavy gray arrows illustrate different nonradiative relaxation processes. The dashed lines indicate midgap states created by oxygen vacancies coordinately unsaturated indium(III) sites and surface hydroxyl groups, respectively.

Supplementary Table 1. The desorption temperature and desorbed quantity of CO_2 from CO_2-TPD profiles.

	rh-250	rh-300	rh-350	rh-400	c-$In_2O_{3-x}(OH)_y$
Desorption temperature (°C)	144.3	137.6	139.9	141.5	157.2
Quantity of CO_2 (mmol g^{-1})	0.041	0.042	0.039	0.040	0.188

Supplementary Table 2. Formation energy of surface vacancies on the rhombohedral $In_2O_{3-x}OH_y$ nanostructure at the (110) direction.

Vacancy site	1	2	3	4	5	6	7	8	9
Energy [eV]	4.94	4.73	4.15	4.22	4.72	4.26	4.22	4.51	4.23

Supplementary Table 3. Triple exponential fitting parameters of the PL decay curves in Supplementary Figure 30.

	τ_1 (ns)	β_1 (%)	τ_2 (ns)	β_2 (%)	τ_3 (ns)	β_3 (%)	τ_{avg} (ns)	χ^2
rh-250	0.97	1.70	49.55	1.1	0.16	97.21	0.17	0.96
rh-300	7.56	0.57	96.44	4.2	0.15	95.24	0.16	1.08
**rh-350**	**11.55**	**0.95**	**11.42**	**7.14**	**0.16**	**91.91**	**0.17**	**1.21**
rh-400	1.05	1.77	41.94	2.84	0.12	95.40	0.12	1.15
c-$In_2O_{3-x}(OH)_y$	1.43	1.51	86.77	5.79	0.15	92.70	0.16	1.18

Supplementary Table 4. Double exponential fitting parameters of the PL decay curves in Supplementary Figure 30.

	τ_1 (ns)	β_1 (%)	τ_2 (ns)	β_2 (%)	τ_{avg} (ns)	χ^2
rh-250	0.012	39.98	0.176	60.02	0.027	1.399
rh-300	0.413	97.08	74.091	2.92	0.425	2.023
rh-350	0.415	94.66	84.923	5.34	0.439	2.171
rh-400	0.387	89.37	101.12	10.63	0.433	1.942
c-$In_2O_{3-x}(OH)_y$	2.075	0.71	0.421	99.29	0.424	2.795
